# p53-dependent induction of P2X7 on hematopoietic stem and progenitor cells regulates hematopoietic response to genotoxic stress

**DOI:** 10.1038/s41419-021-04202-9

**Published:** 2021-10-08

**Authors:** Lin Tze Tung, HanChen Wang, Jad I. Belle, Jessica C. Petrov, David Langlais, Anastasia Nijnik

**Affiliations:** 1 Department of Physiology, McGill University, Montreal, QC Canada; 2 McGill University Research Centre on Complex Traits, McGill University, Montreal, QC Canada; 3 Department of Human Genetics, McGill University, Montreal, QC Canada; 4 McGill University Genome Centre, McGill University, Montreal, QC Canada; 5 Department of Microbiology and Immunology, McGill University, Montreal, QC Canada

**Keywords:** Tumour-suppressor proteins, Transcriptomics, Haematopoietic stem cells

## Abstract

Stem and progenitor cells are the main mediators of tissue renewal and repair, both under homeostatic conditions and in response to physiological stress and injury. Hematopoietic system is responsible for the regeneration of blood and immune cells and is maintained by bone marrow-resident hematopoietic stem and progenitor cells (HSPCs). Hematopoietic system is particularly susceptible to injury in response to genotoxic stress, resulting in the risk of bone marrow failure and secondary malignancies in cancer patients undergoing radiotherapy. Here we analyze the in vivo transcriptional response of HSPCs to genotoxic stress in a mouse whole-body irradiation model and, together with p53 ChIP-Seq and studies in p53-knockout (p53KO) mice, characterize the p53-dependent and p53-independent branches of this transcriptional response. Our work demonstrates the p53-independent induction of inflammatory transcriptional signatures in HSPCs in response to genotoxic stress and identifies multiple novel p53-target genes induced in HSPCs in response to whole-body irradiation. In particular, we establish the direct p53-mediated induction of P2X7 expression on HSCs and HSPCs in response to genotoxic stress. We further demonstrate the role of P2X7 in hematopoietic response to acute genotoxic stress, with P2X7 deficiency significantly extending mouse survival in irradiation-induced hematopoietic failure. We also demonstrate the role of P2X7 in the context of long-term HSC regenerative fitness following sublethal irradiation. Overall our studies provide important insights into the mechanisms of HSC response to genotoxic stress and further suggest P2X7 as a target for pharmacological modulation of HSC fitness and hematopoietic response to genotoxic injury.

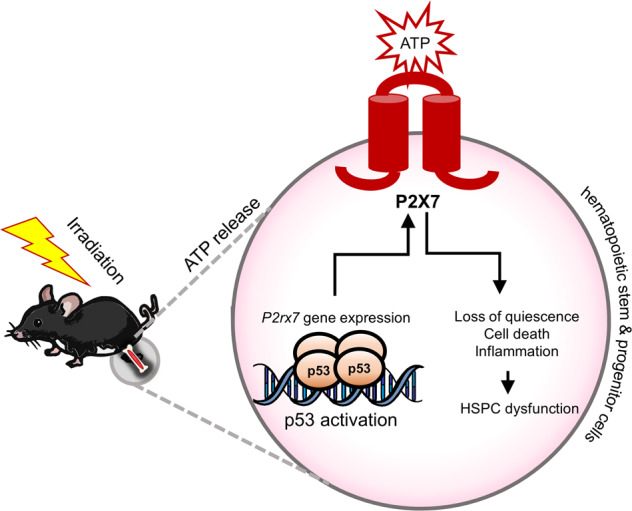

## Introduction

Genotoxic stress is defined as cellular exposure to DNA-damaging agents and the biochemical response of the cells to such damage. Understanding of the specialized stress response mechanisms of tissue stem cells is essential to predict and modulate its complex effects on the organism. Hematopoietic stem cells (HSCs) are the precursors of all blood cells, and genotoxic stress in HSCs can predispose to bone marrow failure and hematologic malignancies [1, 2].

p53 is the major transcriptional regulator of cellular stress responses [3], and its target genes have been characterized in many cell lines [3, 4], fibroblasts [5], macrophages [6], and other cells [7]. These studies identified both the common transcriptional programs of p53 stress response shared by most cells and some cell-type-restricted p53-regulated genes, underlying the unique features of stress response in those cells.

p53 is reported to have specialized functions in HSCs under homeostatic conditions [8], including promoting quiescence by maintaining *Gfi1* and *Ndn* expression [9–11]. In contrast, in response to genotoxic stress, HSCs have a dampened induction of p53-regulated apoptotic genes, resulting in their resistance to apoptosis, as compared with progenitors and mature blood cells [12–14]. However, the p53-regulated transcriptome of HSCs was characterized primarily with microarray studies in mixed stem and progenitor samples [12–14]. Further studies in this area are expected to provide novel insights into the role of p53 in HSCs, and the mechanisms underlying their distinct cell-fate decisions in response to cytotoxic stress.

Irradiation (IR) and genotoxic stress also drive the release of many secreted mediators, signaling the presence of danger and damage to the surrounding tissues [15, 16], and ATP is one of such paracrine danger signals [15–18]. P2X7 is a widely studied receptor for extracellular ATP and a ligand gated cation channel [19]. It is highly expressed by many cells of the blood and immune system, as well as other cell types. Its stimulation induces the release of inflammatory cytokines, production of reactive oxygen species (ROS), activation of proteases followed by shedding of various adhesion molecules, and the induction of cell death [19, 20]. P2X7 is commonly upregulated in hematologic malignancies to act as a modulator of disease progression and antitumor immunity and also a potential drug target for anticancer therapy [21, 22].

HSCs are known to respond to ATP and other P2X7 agonists, with loss of quiescence, mobilization from the niche, biased differentiation toward the myeloid lineage, or proliferative exhaustion [23, 24], and an ectopic overexpression of P2X7 can result in similar phenotypes [25]. Interestingly, P2X7 is highly polymorphic in human and some haplotypes correlate with more efficient granulocyte colony-stimulating factor-induced HSC mobilization [26], further supporting the functional significance of P2X7 on HSCs.

Here we mapped the genome-wide transcriptional response of murine hematopoietic stem and progenitor cells (HSPCs) to whole-body irradiation (WBI) and used p53-knockout mice to categorize the p53-dependent and p53-independent features of the response. We identify many novel p53-regulated genes with potentially interesting roles in HSC biology. In particular, we demonstrate that P2X7-encoding gene (*P2rx7*) is a direct transcriptional target of p53. P2X7 is induced in HSCs and HSPCs in response to WBI via p53-dependent mechanisms and acts as an important regulator of hematopoietic fitness in the response to genotoxic stress.

## Results

### HSPC transcriptional response to IR: goals and methodology

**Fig. 1:**
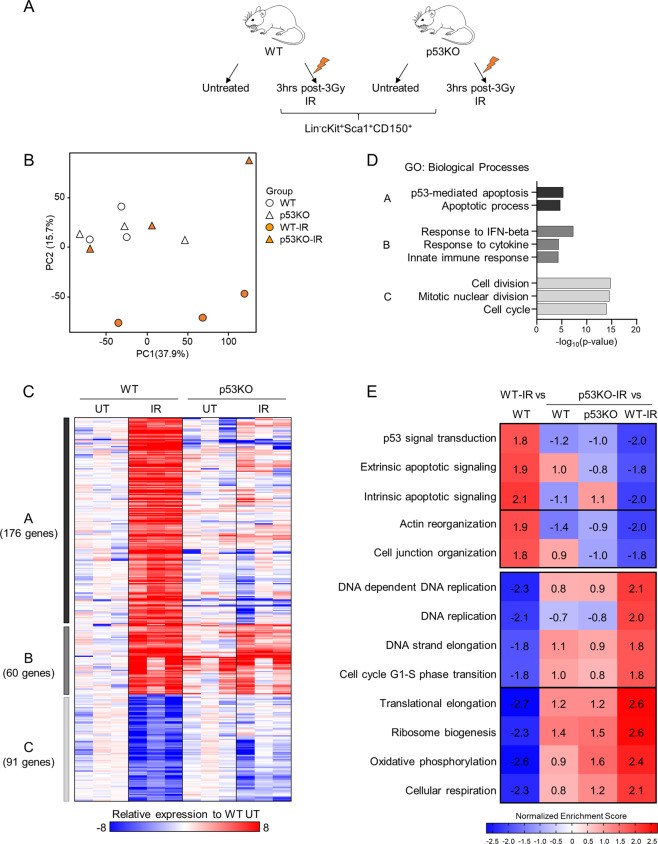
Transcriptional responses of murine HSPCs to whole-body irradiation. **A** Schematic representation of the experimental design and gating strategies. Wild-type C57BL/6 (WT) and *p53*^−/−^ (p53KO) mice were subjected to 3-Gray whole-body irradiation and Lin−cKit^+^Sca1^+^CD150^+^ HSPCs were isolated from the bone marrow 3 h later for RNA-Seq transcriptional profiling. **B** Principal component analysis graph representing the gene expression profiles of each RNA-Seq sample and showing a major transcriptional response to genotoxic stress in WT-IR but not p53KO-IR HSPCs (PC2, 15.7% variability). **C** Heatmap displaying 327 significantly dysregulated genes in WT-IR or p53KO-IR relative to control WT HSPCs. The significance threshold is fold change ≥|2| and false discovery rate (FDR) ≤0.05. Relative expression levels to the average of WT group are used to generate the heatmap. Hierarchical clustering of the genes using Pearson correlation and complete linkage generated three distinct gene clusters. **D** Gene ontology (GO) enrichment analysis on genes from the three gene clusters described in **C**. Top 2–3 enriched biological process GO terms are displayed; the full list is available in Table S1C. **E** Gene set enrichment analysis (GSEA) [83] is performed with 4436 pre-established biological process gene signatures. Heatmap displays the normalized enrichment scores (NES) of upregulated and downregulated p53-dependent HSPC transcriptional signatures in response to irradiation, with NES ≥ | 1.8| in WT-IR versus WT RNA-Seq datasets.

**Fig. 2:**
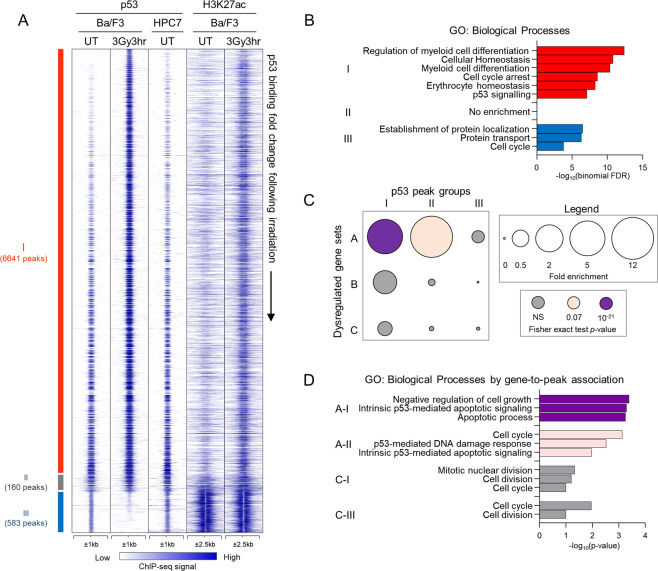
Mapping the genome-wide p53 DNA-binding sites and p53-regulated genes in murine HSPCs in response to whole-body irradiation. **A** Heatmap displays 7384 p53 DNA-binding sites, or peaks, identified from p53 ChIP-Seq in Ba/F3 and HPC7 hematopoietic progenitor cells, with and without irradiation (3 Gy, 3 h). The peaks are ordered based on fold changes in p53-binding intensity following irradiation (IR) and organized into three groups. Group I is composed of peaks with ≥ 1.5 fold increase in p53 binding following IR, Group II is composed of peaks with minimal change in p53 binding (FC ≤ | 1.5 | ), and Group III is composed of peaks with ≥1.5 fold decrease in p53 binding following IR. For the p53 ChIP-Seq, binding intensities ±1 kb around the p53 peak centers are plotted. For the histone H3K27ac ChIP-Seq, binding intensities ±2.5 kb around the p53 peak centers are plotted. H3K27ac ChIP-Seq in Ba/F3 cells was previously described [32]. **B** Gene ontology analysis of genes nearest to p53 peaks, performed using GREAT [37] with basal plus extension option and searching for genes 2 kb upstream, 2 kb downstream, and 200 kb in distal to each p53 peak. The −log10(binomial FDR) values are plotted for each GO term. **C** Association of RNA-Seq and ChIP-Seq data shows dysregulated genes having p53 peaks within 10 kb. The circle size represents fold enrichment of dysregulated genes in the gene-to-peak association compared to random gene sets. The color represents the significance of the fold enrichment, calculated by two-tailed Fisher exact test. The random gene sets consist of 10 sets of 200 genes randomly selected from the total 13,753 expressed genes identified by RNA-Seq. NS not significant. **D** Gene ontology analysis of putative p53-target genes identified as genes showing p53-dependent dysregulation in RNA-Seq data and having p53-binding peaks within 10 kb. The top biological process terms are displayed, and −log10(*p* values) are plotted.

To map the global transcriptional response of HSPCs to IR within their physiological niche and to distinguish the p53-dependent and independent features of this response, we performed RNA sequencing (RNA-Seq) transcriptional profiling on FACS (fluorescence-activated cell sorting)-sorted Lin^−^cKit^+^Sca1^+^CD150^+^ HSPCs from wild-type (WT) and *Tp53*-knockout (p53KO) mice, untreated and at 3 hours after 3 Gy IR (Fig. 1). Additionally, to map p53 DNA-binding sites, p53 chromatin immunoprecipitation sequencing (ChIP-Seq) was performed in multipotent progenitor cells HPC7 and B cell progenitors Ba/F3, both selected for their p53 WT status (Fig. 2) [27, 28]. The resulting datasets were analyzed with the following goals: (I) to characterize the p53-dependent transcriptional response of HSPCs to WBI and to identify the genes under direct transcriptional control of p53; (II) to characterize the p53-independent transcriptional response of HSPCs to WBI; (III) to assess p53 functions in HSPC transcriptional landscape under homeostatic conditions; and (IV) to functionally characterize novel mediators of HSPC response to WBI.

### p53 is the major mediator of classical transcriptional response of HSPCs to IR

RNA-Seq transcriptional profiles from WT, WT-IR, p53KO, and p53KO-IR HSPCs (Fig. 1A) were subjected to dimension reduction analysis. This demonstrated a clear separation between WT-IR and control WT HSPCs, indicating a major transcriptional response to IR (PC2 15.7%, Fig. 1B). In contrast, no separation was observed between p53KO-IR and control WT or p53KO HSPCs, indicating p53 as the major mediator of the HSPC response to IR. Furthermore, there was no separation between the p53KO and WT HSPCs, suggesting that p53 is not a significant regulator of HSPC transcriptional state under homeostatic conditions (Fig. 1B).

Differential gene expression analysis was performed at fold change (FC) ≥2 and false discovery rate (FDR) ≤ 0.05. This identified 327 dysregulated genes in WT-IR relative to control WT HSPCs, including 236 upregulated and 91 downregulated (Fig. 1C and Tables S1A, B). In contrast, only seven genes were significantly dysregulated in p53KO-IR HSPCs, being increased in expression relative to WT, further confirming the key role of p53 in the IR-induced transcriptional response. Hierarchical clustering of the significantly dysregulated genes in at least one condition segregated them into Clusters A–C (Fig. 1C), with Cluster A representing genes upregulated in WT-IR but not in p53KO-IR (176 genes), Cluster B representing genes upregulated in WT-IR and with a trending or significant upregulation in p53KO-IR (60 genes), and Cluster C representing the genes downregulated in WT-IR but not in p53KO-IR (91 genes), all relative to the control WT group. Gene ontology (GO) enrichment analysis was performed for each gene Cluster, to explore the biological processes characterized by the transcriptional signatures. We observed a strong enrichment of GO terms related to “p53 mediated apoptosis” for Cluster A and “cell cycle” for Cluster C (Fig. 1D and Table S1C), indicating the p53-mediated induction of cell cycle arrest and programmed cell death in HSPCs in response to IR. Cluster B, representing the p53-independent transcriptional response of HSPCs to IR, will be discussed below.

Gene set enrichment analysis (GSEA) was applied to characterize the p53-dependent transcriptional response of HSPCs to IR, represented by the transcriptional signatures of WT-IR but not of p53KO-IR relative to control HSPCs (Fig. 1E and Table S1D). This further supported the primary role of p53 in the induction of the transcriptional programs of apoptosis and cell cycle arrest (Fig. 1E, top-middle). The analyses further indicated a p53-mediated repression of anabolic cellular processes, such as “ribosome biogenesis,” “translation,” “oxidative phosphorylation,” and others (Fig. 1E, bottom, and Table S1D), indicating the major role of p53 as a regulator of HSPC metabolic state under conditions of stress.

### p53-binding sites across the genome of hematopoietic progenitor cells

To map p53 DNA binding in hematopoietic progenitors, ChIP-Seq was performed in HPC7 and Ba/F3 hematopoietic progenitor lines and demonstrated a strong concordance in p53-binding sites between the two cells (Fig. 2A and Table S2A). In parallel, the analysis was done in Ba/F3s following IR (3 Gy, 3 h) and the combined data revealed 7384 p53-binding sites (or peaks) (Fig. 2A and Table S2A). Consolidation of the data with previously published p53 ChIP-Seq demonstrated a strong concordance in p53 binding with B cells [29, 30], macrophages [31], and fibroblasts [5] (Fig. S1 and Tables S2A, B), supporting strong conservation of p53 function across cell lineages.

Ordering p53 peaks based on distance to the nearest gene transcription start site (TSS), 16% were classified as gene proximal (≤1 kb to TSS, 1158 sites) and 84% as gene distal (>1 kb to TSS, 6226 sites). p53 peaks were further classified based on change in p53 binding with IR, with the binding increased (6641 peaks), unchanged (160 peaks), or reduced (583 peaks) for Groups I–III peaks, respectively (Figs. 2A and S2A, and Table S2A). ChIP-Seq for an epigenetic mark of transcriptional activation histone H3K27ac, described previously [32], indicated that chromatin activity was enhanced at Group I and unchanged or reduced at Group II–III p53 peaks with IR, respectively (Figs. 2A and S2B), correlating p53 binding and *cis*-regulatory activity at these sites. De novo motif analysis at the p53 peaks identified p53 motif enrichment at Group I–II peaks, suggesting direct p53 binding to DNA at these sites (Fig. S2C). In contrast, no p53 motif enrichment was seen at Group III peaks, suggesting that p53 recruitment at these sites may be indirect. Interestingly, PRDM14 motif was enriched at Group I, SP1 motif at Groups II–III, and NRF1 motif at Group III p53 peaks, suggesting a crosstalk between p53 and these factors in transcriptional regulation, as indicated by previous studies [33–36].

For functional insights into the p53-regulated genes in hematopoietic progenitors, GO analysis was performed on the genes in the vicinity of each p53 peak using GREAT [37]. Genes corresponding to Group I p53 peaks showed strong enrichment of GO terms “signal transduction by p53,” “cell cycle arrest,” and “apoptotic signaling” (Fig. 2B and Table S2C), corresponding to the classical p53 transcriptional program. Surprisingly, Group I genes also showed enrichment of GO terms “myeloid cell differentiation” and “erythroid homeostasis” (Fig. 2B and Table S2C). Indeed, distal p53 binding was observed at several genes critical to hematopoiesis (*Runx1*, *Gfi1b*, *Gata2*, *Gata3*, *Foxo3*, *Sp1*, *Csf1*, and *Kitl*, Table S2D). While some of these p53 sites are seen in previously published p53 ChIP-Seq datasets (Table S2D) [5, 29–31], others appear novel to our data, possibly reflecting specialized p53 functions in hematopoietic progenitors. Nevertheless, the normal expression of these genes in our RNA-Seq indicates that p53 does not regulate their expression in HSPCs, either at steady state or following IR, making the functional significance of p53 binding difficult to infer (Table S2D). No significant enrichment of GO terms was observed for Group II p53 peaks (Fig. 2B), reflecting the small number of p53-binding sites within this group.

### Identification of direct p53-target genes in HSPC response to IR

To identify the genes directly regulated by p53 in the hematopoietic response to IR, our ChIP-Seq and RNA-Seq datasets were consolidated to identify 131 genes that had p53-binding sites in their proximity and were dysregulated in response to IR in WT-IR but not in p53KO-IR HSPCs (Table S3A). Repeating the analysis for each of the dysregulated gene Clusters A–C (Fig. 1C) and for each Group I–III of p53 DNA-binding sites (Fig. 2A), we demonstrated a strong association of the p53-dependent IR-activated Cluster A genes with Group I–II p53-binding sites (Fig. 2C). GO term enrichment analysis on the p53-target genes demonstrated a strong enrichment of GO terms of the classical p53 stress response, including “negative regulation of cell growth,” “apoptotic process,” and “ DNA damage response” (Fig. 2D and Table S3C). These results provide a comprehensive characterization of the p53-regulated genes in murine HSPCs in the physiological response to WBI and will be further explored below for novel findings.

### p53-independent immunological transcriptional signatures in HSPCs in response to IR

**Fig. 3:**
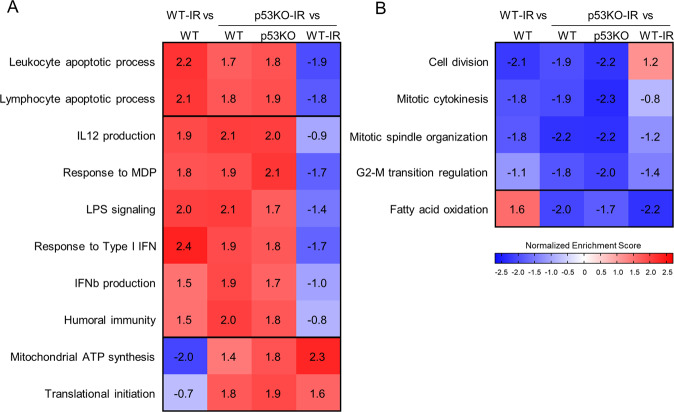
Identification of p53-independent GSEA transcriptional signatures in HSPCs in response to whole-body irradiation. Gene set enrichment analysis (GSEA) is performed with 4436 pre-established biological process gene signatures. Heatmap displays the normalized enrichment scores (NES) of **A** upregulated and **B** downregulated p53-independent transcriptional signatures in murine HSPCs in response to whole-body irradiation, with NES ≥ | 1.8| in the comparison between the p53KO-IR dataset and the control WT and/or p53KO RNA-Seq datasets.

To explore the p53-independent HSPC transcriptional signatures in response to WBI, GSEA was carried out to compare p53KO-IR and control HSPCs, showing that the transcriptional signatures of programmed cell death and G2-M cell cycle arrest were induced in HSPCs in part via p53-independent mechanisms (Fig. 3A, B and Table S1D). GSEA analysis further demonstrated a strong p53-independent induction of immune and inflammatory transcriptional signatures in HSPCs in response to IR, exemplified by upregulated terms “response to IFN,” “IL12 production,” “LPS signaling,” and others (Fig. 3A and Table S1D). This was further supported by the GO term enrichment analysis on the genes upregulated in p53KO-IR HSPCs (Cluster B, Fig. 1C), with enrichment of terms “response to IFNβ,” “response to cytokine,” “innate immune response,” and others (Fig. 1D and Table S1C). We conclude that the p53-independent transcriptional response of HSPCs to IR shares many features of the responses classically induced by immune and inflammatory stimuli.

GSEA also indicated that the transcriptional response of p53KO-IR HSPCs is characterized by upregulation of anabolic processes like “mitochondrial ATP synthesis” and “translational initiation” (Fig. 3A and Table S1D) and by downregulation of “fatty acid oxidation” (Fig. 3B and Table S1D). Importantly, these transcriptional signatures were in strong contrast to WT-IR HSPCs, where we previously noted a global repression of anabolic cellular processes (Fig. 1E and Table S1D). This further highlights the distinct transcriptional regulation of the metabolic response to genotoxic stress in WT and p53KO HSPCs.

### p53 and HSPC transcriptional landscape under homeostatic conditions

**Fig. 4:**
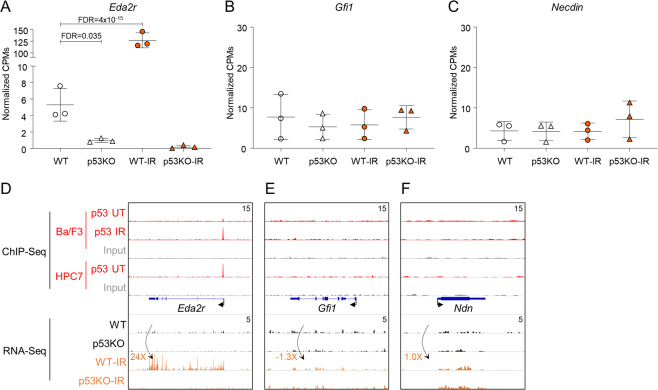
Characterization of the p53-regulated genes in HSPCs under homeostatic conditions. **A**–**C** Normalized counts per million (CPM) expression of *Eda2r*, *Gfi1*, and *Ndn* genes from the RNA-Seq of murine WT and p53KO HSPCs with and without whole-body irradiation. **D**–**F** Genomic snapshots of *Eda2r*, *Gfi1*, and *Ndn* genes. ChIP-Seq tracks of p53 in untreated (UT) and irradiated (IR) hematopoietic progenitor cells Ba/F3 and HPC7, and input DNA from the same cells are shown in the top five lanes. The gene feature track is shown in the middle. RNA-Seq tracks of the summation of gene expression in each group are shown in the bottom four lanes. The maximum data range of each track is indicated at the top-right corner of the track.

Comparison of untreated WT and p53KO HSPCs revealed *Eda2r* as the only differentially expressed gene (FC = 0.18, FDR = 0.035, Fig. 4A), indicating that p53 maintains *Eda2r* expression in HSPCs under homeostatic conditions. *Eda2r* expression was also p53 regulated post-IR, with *Eda2r* induced in WT-IR (FC = 24, FDR = 4 × 10^−15^) but not p53KO-IR HSPCs (Fig. 4A). ChIP-Seq confirmed steady-state p53 binding at *Eda2r* promoter, with increased binding post-IR (Fig. 4D). In contrast, we did not observe dysregulation of *Gfi1* or *Ndn* genes in p53KO HSPCs, either at steady state or following IR and did not detect p53 binding at these genes (Fig. 4B, C, E, F), in contradiction to previous reports [9–11].

### Novel mediators of HSPC response to IR

To identify novel p53-target genes, we screened our list of 131 p53-regulated genes (Figs. 1 and 2 and Table S3A), against the list of KEGG mmu04115 “p53 signaling pathway” genes ([38]; https://www.genome.jp/kegg/kegg1.html) and similar lists from 14 previous studies in other cells, reviewed by Fischer et al. [4]. Additionally, we performed an automated PubMed search for publications that include each gene name together with the term “p53” in the title or the abstract of an article. Based on these criteria, we identified 38 potentially novel p53-target genes in our data (Table S3B and Fig. S2D). We further focused on “druggable” genes, with potential for pharmacological modulation of HSPC response to genotoxic stress, by searching the DGIdb database (Table S3D) [39]. Based on this, *P2rx7* was identified as a novel p53-target gene, encoding a highly “druggable” protein [19].

### *P2rx7* is a p53-target gene in HSPCs

**Fig. 5:**
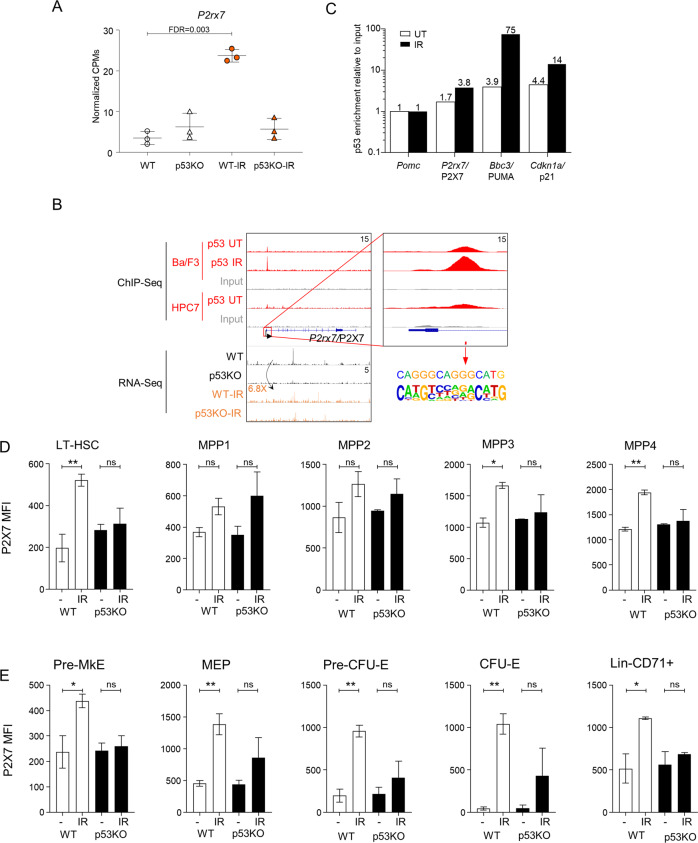
P2rx7 as a putative novel p53-target gene in HSPC response to irradiation. **A** Normalized counts per million (CPM) expression of *P2rx7* gene in WT and p53KO HSPCs with and without whole-body irradiation. **B** Genomic snapshots of *P2rx7*. ChIP-Seq tracks of p53 in untreated (UT) and irradiated (IR) hematopoietic progenitor cells Ba/F3 and HPC7, and input DNA from the same cells are shown on the top five lanes. The gene feature track is shown in the middle. RNA-Seq tracks of the summation of expression in each group are shown in the bottom four lanes. The maximum data range of each track is indicated at the top-right corner of the track. Red box highlights the p53 consensus binding motif identified at the promoter of the *P2rx7* gene. **C** Enrichment of p53 at the promoter of *P2rx7* gene, validated with ChIP-qPCR in Ba/F3 hematopoietic progenitor cells. p53-regulated genes *Bbc3/*PUMA and *Cdkn1a/*p21 served as positive controls. Data presented are from one experiment and was reproduced in three independent experiments. **D**, **E** P2X7 expression on HSCs and hematopoietic progenitor cells of WT and p53KO mice, with and without whole-body irradiation (3 Gy, 6 h), analyzed by flow cytometry; mean fluorescence intensity of the P2X7 staining is plotted for each cell population. Data are from 3 to 4 mice per genotype per condition, consolidated from 2 to 3 independent experiments; bars represent means and standard errors on the mean (SEM); statistical analysis by ANOVA followed by Sidak’s multiple comparison post hoc test to compare WT versus WT-IR and p53KO versus p53KO-IR datasets; *p < 0.05, **p < 0.01, or ns not significant. Cells are gated as: **D** Lin^−^cKit^+^Sca1^+^ followed by CD150^+^CD48^−^CD34^−^Flt3^−^ for long-term HSCs (LT-HSCs), CD150^+^CD48^−^CD34^+^Flt3^−^ for MPP1, CD150^+^CD48^+^CD34^+^Flt3^−^ for MPP2, CD150^−^CD48^+^CD34^+^Flt3^−^ for MPP3, and CD150^−^CD48^+^CD34^+^Flt3^+^ for MPP4; **E** Lin^−^cKit^+^Sca1^−^ followed by CD41^−^CD16/32^−^CD150^+^CD105^−^ for pre-megakaryocyte erythroid progenitors (pre-MkE), CD34^−^CD16/32^−^ megakaryocyte erythroid progenitors (MEP), CD41^−^CD16/32^−^CD150^+^CD105^+^ for pre-CFU-E erythroid progenitors, and CD41^−^CD16/32^−^CD150^−^CD105^+^ for CFU-E erythroid progenitors; or as Lin^−^CD71^+^ for the more mature erythroid precursor cells.

P2X7 is a purinergic receptor for extracellular ATP [19], and its role in p53-dependent hematopoietic response to genotoxic stress has not been characterized. We demonstrate a p53-dependent induction of *P2rx7* expression in HSPCs with WBI (Fig. 5A), and p53 binding to the *P2rx7* gene promoter (Fig. 5B, C). We further demonstrate a p53-dependent upregulation of P2X7 protein by flow cytometry on long-term HSCs and on some multipotent, myeloid, and erythroid progenitors (Figs. 5D, E and S3A, B). P2X7 induction was most pronounced for erythroid progenitors, as exemplified by CFU-E cells that express minimal P2X7 at homeostatic conditions and strongly upregulate P2X7 with WBI (Figs. 5E and S3A). In contrast, we observed no induction of P2X7 with IR on mature B cells or erythrocytes (data not shown). Overall, we establish *P2rx7* as a p53-target gene in murine HSCs and HSPCs in the context of WBI.

### P2X7 in hematopoiesis at homeostatic conditions

We assessed P2X7 functions in hematopoiesis under homeostatic conditions, using P2X7-knockout mice (P2X7-KO) [40]. We observed no significant defects, with normal numbers of HSPCs and all mature blood cells (Figs. S4–S9). We observed a mild but significant increase in megakaryocyte erythroid progenitors (Fig. S4A–C) and B cell precursors (Fig. S5) in P2X7-KO bone marrow; and although corresponding mature cells were unchanged (Figs. S6 and S9), this may reflect some increase in the capacity for regeneration of these cells under stress. We further assessed P2X7-KO HSCs in a chimeric model, with bone marrow from P2X7-KO or WT mice mixed with CD45.1^+^ competitor marrow in a 1:1 ratio, and reconstituted into two independent groups of WT B6-SJL lethally irradiated recipients (Fig. S10A). No differences were observed in the contribution of P2X7-KO and WT HSCs to hematopoietic lineages of the recipient mice (Fig. S10B–E).

### P2X7 in hematopoietic response to genotoxic stress

**Fig. 6:**
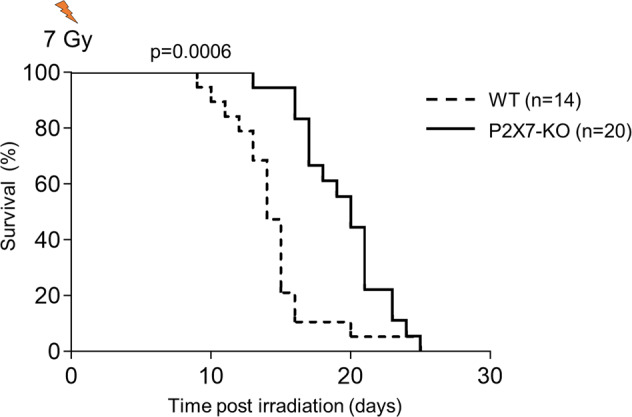
Role of P2X7 in hematopoietic response to acute irradiation. P2X7-KO and WT mice were subjected to whole-body irradiation at the dose of 7 Gy and mouse survival was monitored. A significant delay in the lethality of P2X7-KO mice relative to WT control mice is observed. Data are from 14 WT and 20 P2X7-KO mice, including males and females sex matched between the genotypes; data are consolidated from two independent experiments; statistical analyses using Log-rank Mantel–Cox test, *p* = 0.0006.

To assess P2X7 in the regulation of hematopoietic response to genotoxic stress, P2X7-KO and WT mice were irradiated at 7 Gy and their survival was monitored. Importantly, at this dose lethality is mediated by hematopoietic failure and can be rescued through bone marrow transplantation. We observed a significant delay in the lethality of P2X7-KO relative to WT mice (Fig. 6), establishing P2X7 as a mediator of hematopoietic response to acute IR.

**Fig. 7:**
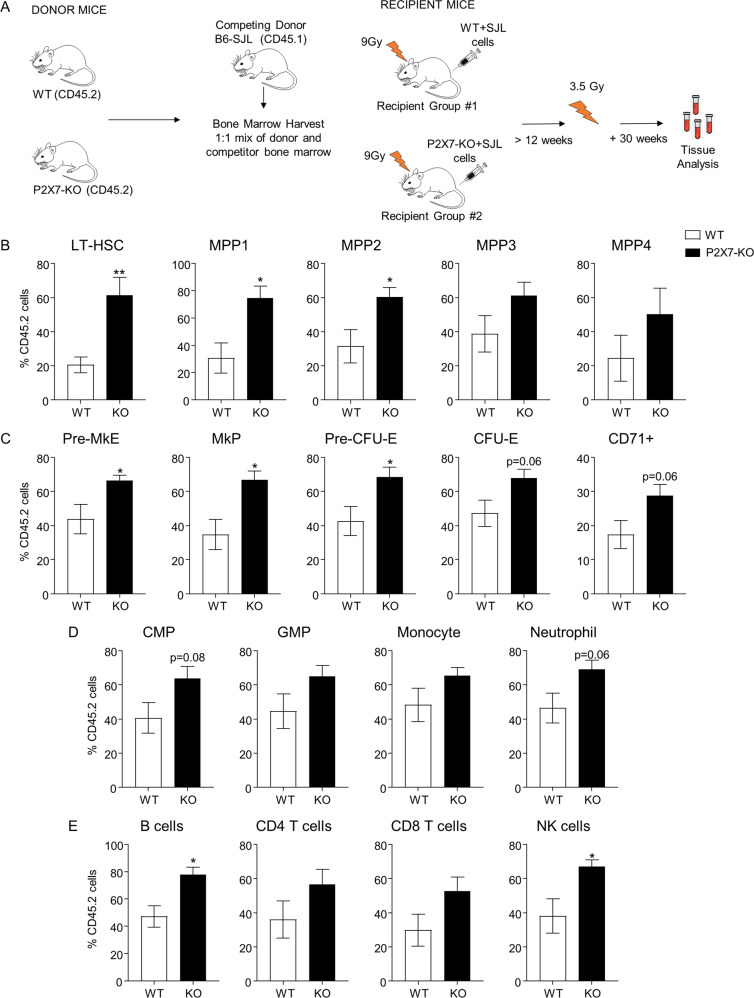
Assessing the role of P2X7 in the long-term HSC response and resistance to sublethal irradiation, using a competitive bone marrow transplantation model. Bone marrow from P2X7-KO or WT mice was mixed with CD45.1-marked competitor bone marrow in a 1:1 ratio and reconstituted into two independent groups of lethality irradiated wild-type B6-SJL recipients. Following full reconstitution, the recipient mice were subjected to a sublethal irradiation at 3.5 Gy, allowed to recover over a 30-week period, and subsequently analyzed for the relative contribution of P2X7-KO and WT HSCs to hematopoietic lineages. The frequency of CD45.2^+^CD45.1^−^ cells within each cell population is presented. Data are from 3 to 5 mice per group; bars represent means and standard errors on the mean (SEM); statistical analysis by Student’s *t* test; *p < 0.05, **p < 0.01, or not significant if significance is not indicated. **A** Schematic representation of the mouse to mouse competitive bone marrow transplantation study. **B** Relative contribution of P2X7-KO and WT cells to the HSC and multipotent progenitor (MPP) cell populations in mouse bone marrow. Cells are gated as: Lin^−^cKit^+^Sca1^+^ followed by CD150^+^CD48^−^CD34^−^Flt3^−^ for LT-HSCs, CD150^+^CD48^−^CD34^+^Flt3^−^ for MPP1, CD150^+^CD48^+^CD34^+^Flt3^−^ for MPP2, CD150^−^CD48^+^CD34^+^Flt3^−^ for MPP3, and CD150^−^CD48^+^CD34^+^Flt3^+^ for MPP4. **C** Relative contribution of P2X7-KO and WT cells to the erythroid and megakaryocyte progenitor cell populations in the mouse bone marrow. Cells are gated as Lin^−^cKit^+^Sca1^−^ followed by CD41^−^CD16^/^32^−^CD150^+^CD105^−^ for pre-megakaryocyte erythroid progenitor (pre-MkE), CD16/32^−^CD150^+^CD41^+^ for megakaryocyte progenitor (MkP), CD41^−^CD16/32^−^CD150^+^CD105^+^ for pre-CFU-E erythroid progenitor, CD41^−^CD16/32^−^CD150^−^CD105^+^ for CFU-E erythroid progenitor, and CD71^+^ for the more mature erythroid precursor cells. **D** Relative contribution of P2X7-KO and WT cells to the myeloid leukocyte cell lineage in the mouse bone marrow. Cells are gated as Lin^−^cKit^+^Sca1^−^ followed by CD34^+^CD16^/^32^−^ for the common myeloid progenitor (CMP) and CD34^+^CD16/32^+^ for the granulocyte monocyte progenitor (GMP), CD11b^+^Ly6G^−^Ly6C^+^ for monocytes, and CD11b^+^Ly6G^+^Ly6C^−^ for neutrophils. **E** Relative contribution of P2X7-KO and WT cells to lymphoid cell populations in the mouse spleen. Cells are gated as B220^+^ for B cells, CD3^+^CD4^+^ and CD3^+^CD8^+^ for T cells, and CD3^-^NK1.1^+^ for NK cells.

To assess P2X7 in long-term HSC response to sublethal IR, competitive bone marrow chimeras were set-up as previously. Following full reconstitution, the recipient mice were irradiated at 3.5 Gy, allowed to recover over 30 weeks for full regeneration of hematopoiesis from the HSC compartment, and analyzed for the relative contribution of P2X7-KO and WT HSCs to hematopoietic lineages (Fig. 7A). We observed an enhanced contribution of P2X7-KO cells to the bone marrow pools of HSCs (Figs. 7B and S11) and some multipotent, erythroid, and megakaryocyte progenitors (Fig. 7B, C). P2X7-KO cells also showed stronger contribution to B and natural killer cell populations and a trend toward enhanced reconstitution of neutrophils (Fig. 7D, E). These findings demonstrate the cell intrinsic role of P2X7 in the regulation of HSC fitness in response to IR.

## Discussion

In summary, we have mapped the genome-wide transcriptional response of murine HSPCs to IR and, using p53 ChIP-Seq and p53-knockout mice, categorized the p53-dependent and p53-independent features of this response. We identified multiple novel p53-regulated genes with potentially interesting roles in HSC biology. We further established *P2rx7* as a novel p53-target gene in HSPCs and a regulator of HSC fitness in response to IR.

While previous studies analyzed HSC transcriptional response to WBI [41], the characterization of the p53-dependent and p53-independent branches of this response is novel to our work. Here we describe potent p53-independent inflammatory signatures in HSPCs in response to WBI. Importantly, HSCs are highly responsive to inflammation [42], with interferons and other mediators driving HSC loss of quiescence, proliferative exhaustion, or apoptosis [43, 44]. We therefore propose that the inflammatory transcriptional response of HSPCs may contribute to acute IR-induced hematopoietic failure and the long-term deleterious effects of radiotherapy on hematopoiesis [15, 45].

The p53-independent transcriptional responses of HSPCs may reveal novel approaches to target carcinogenic hematopoietic cells with p53 mutations [46, 47]. In particular, we show that the pro-inflammatory transcriptional response of HSPCs to IR is independent of p53. Synergies between cancer radiotherapy and immunotherapy are increasingly recognized, as radiotherapy can stimulate antitumor immunity [48–50]. The growing understanding of the crosstalk between genotoxic and inflammatory stress pathways, specifically in HSPCs, may suggest new approaches to protect normal or target leukemic HSPCs through optimized combinations of immunotherapy and radiotherapy. Similarly, the distinct metabolic transcriptional regulation in WT and p53KO HSPCs in response to IR, shown here, may also yield novel strategies for the targeting of p53-deficient HSPCs.

We characterize p53 functions in transcriptional regulation under homeostatic conditions, via comparison of WT and p53KO HSPCs (LKS CD150^+^). Previous studies indicated p53 as a regulator of *Gfi1* and *Ndn* expression in HSPCs under homeostatic conditions, based on microarray analyses in less pure HSPCs (LKS) and in an *Elf4*-deficient mouse model [9–11]. These technical differences may account for the fact that we do not observe p53-mediated regulation of *Gfi1* and *Ndn* in our study. In contrast, we report p53-dependent regulation of *Eda2r* in HSPCs both at steady state and following IR. While *Eda2r* is a known p53-regulated gene in hair follicles [51, 52] and colorectal and ovarian tumors [53–55], its role in HSPC physiology is unexplored and merits further investigation. Additionally, we identify p53 binding in the vicinity of genes encoding critical regulators of hematopoiesis, although their expression was not p53 regulated either at steady state or following IR (Table S2D). Polymorphisms in the p53-binding region of *Kitl* were implicated in cancer susceptibility in human [56], and p53 binding at *Kitl* locus regulates coat pigmentation in mice [57]. We suggest that p53 binding at the genes encoding critical regulators of hematopoiesis may be functionally significant in other physiological settings, to be explored in future work.

We identified many novel p53-target genes in HSPCs (Table S3B), to be characterized in future work, while here *P2rx7* was selected for further functional analyses. WBI drives the release of ATP into the extracellular milieu [15–18], and we propose that, together with the p53-driven induction of P2X7 on HSPCs, this contributes to the depletion of stressed and damaged HSPCs via paracrine mechanisms. As *P2rx7* was not previously characterized as a p53-target gene, this effect is likely in part cell type specific, and there are indeed other specialized mechanisms regulating *P2rx7* expression, as, for example, the induction of *P2rx7* by C/EBPβ in intestinal epithelial cells, by Sp1 in neurons and astrocytes [58, 59], and by Tet1 and Tet2 in mesenchymal stem cells [60]. Interestingly, in our work the p53-driven P2X7-induction is most pronounced on HSCs and erythroid progenitors, suggesting specialized roles of P2X7 in the stress response of these cells. While our study focuses on WBI, the role of p53-dependent P2X7 induction in HSC response to other stresses remains to be explored.

The mechanisms mediating the deleterious effects of P2X7 on HSPCs remain to be further addressed. Strong induction of P2X7 on HSPCs, together with the protective effects of P2X7 deficiency in competitive HSC transplantation models, suggest that cell-intrinsic mechanisms are involved. IR is known to promote the release of P2X7-agonist ATP [15–18], and P2X7 stimulation or overexpression can drive HSC mobilization from their niche, differentiation, and proliferative exhaustion [23–25], with prolonged stimulation leading to the formation of nonselective transmembrane pores and cell death [20]. Similarly, direct stimulation of P2X receptors on erythroid progenitors can drive ROS production and apoptosis [61]. While such P2X7-driven responses likely have a physiological role to eliminate stressed and damaged HSPCs, they can also contribute to hematopoietic dysfunction and under acute conditions result in lethal hematopoietic failure. It is also interesting to note that P2X7 stimulation and the resulting potassium efflux are upstream of inflammasome activation [62]. With the growing understanding of inflammasome functions in genotoxic stress responses [63] and in HSPCs [64], the crosstalk between the p53-driven P2X7 expression, inflammasome activation, and loss of HSPC fitness will need to be explored.

The protective effects of P2X7 deficiency in IR-driven hematopoietic failure and in HSC maintenance following sublethal IR, shown here, suggest P2X7 as a drug target for modulation of HSC responses to genotoxic stress. Importantly, relevant P2X7 antagonists are available and have shown efficacy in rodent models of inflammatory, neurological, and other conditions [19]. AZD9056 has also undergone clinical trials, showing efficacy in Crohn’s disease, but not rheumatoid arthritis or chronic obstructive pulmonary disease [65–67]. Importantly, the loss or inhibition of P2X7 was also shown to repress the self-renewal of leukemia-initiating cells and to inhibit leukemogenesis in acute myeloid leukemia mouse models [21, 22, 68], providing further rationale to explore P2X7 antagonists as part of cancer therapy regimens. Whether P2X7 antagonists can also offer protection against the off-target toxicities of radiotherapy remains to be further explored. Interestingly, *P2rx7* gene is polymorphic in human [26], and our study suggests that this may impact the hematopoietic response to genotoxic stress. p53 activation is also a feature of several bone marrow failure disorders [69–71], and whether this promotes P2X7 induction to affect hematologic pathology is at present unknown. These and other implications of our findings to human hematopoiesis in a clinical setting are to be explored in future work.

## Materials and methods

### Mouse lines

WT, P2X7-KO, and p53-KO mice, all on the C57BL/6J genetic background, were obtained from The Jackson Laboratory (JAX002101, JAX005576, and JAX000664) [40], and maintained under specific pathogen-free conditions. Adult mice of both sexes were used in the experiments and always age and sex matched across all the experimental groups. The number of mice used in each experiment and experimental repeats are presented in the figure legends; no specific statistical methods were used to select samples sizes; no samples/animals were excluded from the analyses; no randomization was used to allocate samples/animals to experimental groups; and the investigator was not blinded to the group allocation of samples/animals. Genotypes of all animals were validated with genomic PCR, using DreamTaq DNA Polymerase (Thermo Fisher Scientific) and primers from IDT Technologies. Both male and female mice were used in the study and sex matched across the experimental groups.

### Mouse WBI

Mouse WBI was performed in a RS2000 irradiator (Rad Source), and the mice were kept on neomycin in drinking water (2 g/L, BioShop) for 3 weeks following the procedure.

### Mouse bone marrow transplantation

For competitive bone marrow transplantations, recipient WT B6.SJL-PtprcaPepcb/Boy (JAX002014, congenic for CD45.1) mice were irradiated with 2 doses of 4.5 Gy, delivered 3 h apart, in a RS2000 irradiator (Rad Source). Donor cells were delivered via intravenous injection in sterile phosphate-buffered saline (PBS). The mice were kept on neomycin in drinking water (2 g/L, BioShop) for 3 weeks and analyzed at >20 weeks post-reconstitution.

### Cell culture

Murine B cell progenitor cell line Ba/F3 (DSMZ, ACC 300) was maintained at 0.5–2 × 10^6^ cells/mL in RPMI-1640 (Wisent) with 10% fetal calf serum (FCS, Wisent), 2 mM L-Glutamine, 100 μg/mL streptomycin, 100 U/mL penicillin (Wisent), and 5% WEHI conditioned media as the source of interleukin-3. Multipotent hematopoietic progenitor cells HPC7, derived from murine ES-cells via constitutive expression of LIM-homeobox gene LH2, were provided by Professor Leif Carlsson (Umea Center for Molecular Medicine, Umea, Sweden) [27] and previously extensively characterized [72]. HPC7 cells were cultured at 0.5–2 × 10^6^ cells/mL in IMDM (Life Technologies), with 10% FCS (Gibco, Life Technologies), 100 μg/mL streptomycin, 100 U/mL penicillin (Wisent), 7.48 × 10^−5^ M MTG (M6145, Sigma-Aldrich), and 100 ng/mL murine stem cell factor (Shenandoah Biotechnology, Cedarlane).

### RNA sequencing

The protocols were as previously described [28, 32]. Briefly, RNA was isolated using the MagMAX Total RNA Kit (Ambion, Life Technologies) and quality assessed using Bioanalyzer RNA Pico chips (Agilent). rRNA depletion and library preparation were performed using the SMARTer Stranded RNA-Seq Kit (Takara Clontech). The libraries were sequenced on an Illumina HiSeq 2500 sequencer in paired-end 50 bp configuration. The high quality of sequence reads was confirmed using the FastQC tool (Babraham Bioinformatics), and low-quality bases were trimmed from read extremities using Trimmomatic v.0.33 [73]. Reads were then mapped to the mouse UCSC mm9 reference assembly using TopHat v2.0.9 in conjunction with Bowtie 1.0.0 algorithm [74–76]. Gene expression was quantified by counting the number of uniquely mapped reads with featureCounts using default parameters [77]. We retained genes that had an expression level of minimum 5 counts per million reads in at least three of the samples and performed quantile normalization with the preprocessCore package to remove batch effects [78]. Dimension reduction analysis was performed using the principal component analysis method [79]. TMM normalization and differential gene expression analyses were conducted using the edgeR Bioconductor package version 3.20.9 [80]. Pairwise comparisons were performed between samples across different mouse genotypes. Genes with changes in expression ≥|2| folds and Benjamini–Hochberg adjusted *p* values ≤0.05 were considered significant. For data visualization in Integrative Genomics Viewer (IGV) [81], replicates with the same genotype were combined, and bigwig files were generated using a succession of genomeCoverageBed and wigToBigWig tools and scaled per one million exon-mapped reads. GO and disease ontology enrichment analyses on differentially expressed gene clusters were performed with DAVID Bioinformatics Resources 6.8 [82], and GSEA was performed in command-line using MSigDB v6.0 with default configuration and permutation within gene sets [83]. Automated PubMed search for publications that include gene name together with the term “p53” in the title or the abstract of an article was performed using Entrez Direct tool (https://www.ncbi.nlm.nih.gov/books/NBK179288). We searched for “druggable” genes using the DGIdb database (https://www.dgidb.org/).

### Chromatin immunoprecipitation

ChIP was performed as described previously [28, 32], with minor modifications. Briefly, cells were fixed with 1% formaldehyde in the culture media for 10 min at room temperature, followed by addition of 0.125 M of glycine to stop fixation. Nuclei were extracted with 5 min lysis in 0.25% Triton buffer (10 mM Tris-HCl pH8, 10 mM EDTA, 0.5 mM EGTA), followed by 30 minutes in 200 mM NaCl buffer (10 mM Tris-HCl pH8, 1 mM EDTA, 0.5 mM EGTA). Nuclei were resuspended in sonication buffer (10 mM Tris pH8, 140 mM NaCl, 1 mM EDTA, 0.5 mM EGTA, 0.5% sodium dodecyl sulfate (SDS), 0.5% Triton X-100, 0.05% NaDOC) and sonicated for 14 cycles of 30 s with a digital sonifier (Branson Ultrasonics) at 80%, with 30 s rest in cooled circulating water. Beads were prepared overnight with 40 μL of Dynabeads Protein G (Invitrogen, Life Technologies) conjugated to 5 μg of antibody against p53 (1C12, Cell Signaling Technology) or H3K27ac (ab4729, Abcam). Immunoprecipitation was performed by overnight incubation of antibody–bead matrices with sheared chromatin from the equivalent of 5 × 10^6^ cells. Immune complexes were washed sequentially for 2 min at room temperature with 1 mL of the following buffers: wash B (1% Triton X-100, 0.1% SDS, 150 mM NaCl, 2 mM EDTA, and 20 mM Tris-HCl, pH 8), wash C (1% Triton X-100, 0.1% SDS, 500 mM NaCl, 2 mM EDTA, and 20 mM Tris-HCl, pH 8), wash D (1% NP-40, 250 mM LiCl, 1 mM EDTA, and 10 mM Tris-HCl, pH 8), and TEN buffer (50 mM NaCl, 10 mM Tris-HCl, pH 8, and 1 mM EDTA). The samples were de-crosslinked by overnight incubation at 65 °C in 1% SDS buffer (50 mM Tris-HCl pH8, 10 mM EDTA). Following RNaseA and Proteinase K enzymatic treatments, ChIP DNA was purified using the Qiaquick PCR Cleanup Kit (Qiagen).

### ChIP–quantitative PCR (qPCR)

ChIP enrichment was quantified on a StepOnePlus qPCR instrument with Power SYBR Mastermix (Applied Biosystems, Life Technologies) and primers provided in Supplemental Table S4 (IDT Technologies). All *C*_T_ values were normalized to those of the pro-opiomelanocortin (*Pomc*) gene, which serves as a negative binding region. Enrichment was calculated relative to input DNA.

### ChIP sequencing

ChIP-Seq libraries were prepared using the Illumina TruSeq Kit and sequenced on an Illumina HiSeq 2500 sequencer, with input DNA from the same cells sequenced as negative control. The reads were mapped to the UCSC mouse mm9 reference genome with Bowtie 1.0.0 [75], and p53-binding sites were identified using peak detection algorithm MACS1.4 [84], with comparisons for read enrichment against control input DNA from the same cells. Normalized sequence read density profiles (bigwig) were generated with Homer tool [85] and visualized with IGV [81]. GO and disease ontology enrichment analyses on genes associated with p53 ChIP-Seq binding clusters were performed on GREAT 4.0.4 [37] with Basal plus extension option.

### ChIP-Seq and RNA-Seq data consolidation

Full gene annotations were obtained from UCSC mouse mm9 reference genome. An in-house Python script was developed to load the genomic locations of ChIP-Seq binding sites and RNA-Seq dysregulated genes and search for gene TSSs within a specific distance to each ChIP-Seq binding site. RNA-Seq and ChIP-Seq datasets acquired and described in our study have been deposited into the NCBI public database and are available under GEO Submission GSE171697. Input ChIP-Seq are submitted in GSE150667 and previously described [32].

### Cell sorting

Cell sorting protocols were as previously described [28, 32]. Briefly, bone marrow was flushed in PBS supplemented with 0.1% bovine serum albumin and 2 mM EDTA, filtered through 40 μm cell strainers, and subjected to red blood cell lysis in ACK buffer (0.15 M NH_4_Cl, 10 mM KHCO_3_, 0.1 mM EDTA). The samples were stained with biotin anti-mouse Lineage Panel (BioLegend), PE-Cy7 cKit (clone 2B8, BioLegend), APC-Cy7 Sca-1 (D7, BioLegend), FITC CD48 (HM48-1, eBioscience), APC CD150 (TC15-12F12.2, BioLegend), PE Flt3 (A2F10, eBioscience), Brilliant Violet 421 CD34 (RAM34, BD Biosciences), and streptavidin-PECy5 (BioLegend). Cell sorting was performed on FACSAria, with the FACS Diva software (BD Biosciences).

### Flow cytometry

Cell suspensions of mouse tissues were prepared in RPMI-1640 (Wisent) with 2% (v/v) FCS, 100 μg/mL streptomycin and 100 U/mL penicillin (Wisent). The cells were stained for surface markers in PBS with 2% FCS for 20 min on ice, using antibodies listed in Supplemental Tables S5 and S6. Viability Dye eFluor® 506 (eBioscience) was used to discriminate dead cells. Annexin V PeCy7 (eBioscience) was used for detection of apoptotic cells. Compensation was performed with BD™ CompBeads (BD Biosciences). The data were acquired on BD Fortessa and analyzed with the FACS Diva (BD Biosciences) or the FlowJo (Tree Star) software.

### Mouse hematology

Hematology analysis of mouse blood was performed by the Diagnostics Laboratory of the McGill Comparative Medicine Animal Resources Centre (CMARC), as previously described [32].

### Statistics

Statistical analyses used Prism 7.01 (GraphPad Inc.), with Student’s two-tailed *t* test for two datasets and analysis of variance for multiple comparisons; *p* < 0.05 was considered significant. Further information on the statistical analyses is provided above for RNA-Seq and ChIP-Seq data and in the figure legends for other datasets.

## Supplementary information

Supplemental Materials

Supplemental Table S1

Supplemental Table S2

Supplemental Table S3

## Data Availability

RNA-Seq and ChIP-Seq datasets acquired and described in our study have been deposited into the NCBI public database and are available under GEO Submission GSE171697. Input ChIP-Seq are submitted in GSE150667 and previously described [32]. All other data are either presented in the manuscript or available from the corresponding author upon request.

## Code availability

The bioinformatics software and their original codes are referenced in the “Methods” section. The specific instructions for using the software are available upon request.

## References

[1] Wang Y, Probin V, Zhou D (2006). Cancer therapy-induced residual bone marrow injury-mechanisms of induction and implication for therapy. Curr Cancer Ther Rev.

[2] Dracham CB, Shankar A, Madan R (2018). Radiation induced secondary malignancies: a review article. Radiat Oncol J.

[3] Li M, He Y, Feng X, Huang J (2012). Genome-wide studies of the transcriptional regulation by p53. Biochim Biophys Acta.

[4] Fischer M (2017). Census and evaluation of p53 target genes. Oncogene.

[5] Kenzelmann Broz D, Spano Mello S, Bieging KT, Jiang D, Dusek RL, Brady CA (2013). Global genomic profiling reveals an extensive p53-regulated autophagy program contributing to key p53 responses. Genes Dev.

[6] Lowe JM, Menendez D, Bushel PR, Shatz M, Kirk EL, Troester MA (2014). p53 and NF-kappa B coregulate proinflammatory gene responses in human macrophages. Cancer Res.

[7] Li YW, Liu J, McLaughlin N, Bachvarov D, Saifudeen Z, El-Dahr SS (2013). Genome-wide analysis of the p53 gene regulatory network in the developing mouse kidney. Physiol Genomics.

[8] Liu Y, Elf SE, Asai T, Miyata Y, Liu Y, Sashida G (2009). The p53 tumor suppressor protein is a critical regulator of hematopoietic stem cell behavior. Cell Cycle.

[9] Liu Y, Elf SE, Miyata Y, Sashida G, Huang G, Di Giandomenico S (2009). p53 regulates hematopoietic stem cell quiescence. Cell Stem Cell.

[10] Kubota Y, Osawa M, Jakt LM, Yoshikawa K, Nishikawa S (2009). Necdin restricts proliferation of hematopoietic stem cells during hematopoietic regeneration. Blood.

[11] Asai T, Liu Y, Di Giandomenico S, Bae N, Ndiaye-Lobry D, Deblasio A (2012). Necdin, a p53 target gene, regulates the quiescence and response to genotoxic stress of hematopoietic stem/progenitor cells. Blood.

[12] Mohrin M, Bourke E, Alexander D, Warr MR, Barry-Holson K, Le Beau MM (2010). Hematopoietic stem cell quiescence promotes error-prone DNA repair and mutagenesis. Cell Stem Cell.

[13] Labi V, Erlacher M, Krumschnabel G, Manzl C, Tzankov A, Pinon J (2010). Apoptosis of leukocytes triggered by acute DNA damage promotes lymphoma formation. Genes Dev.

[14] Beerman I, Seita J, Inlay MA, Weissman IL, Rossi DJ (2014). Quiescent hematopoietic stem cells accumulate DNA damage during aging that is repaired upon entry into cell cycle. Cell Stem Cell.

[15] Mavragani IV, Laskaratou DA, Frey B, Candeias SM, Gaipl US, Lumniczky K (2016). Key mechanisms involved in ionizing radiation-induced systemic effects. A current review. Toxicol Res.

[16] Di Maggio FM, Minafra L, Forte GI, Cammarata FP, Lio D, Messa C (2015). Portrait of inflammatory response to ionizing radiation treatment. J Inflamm.

[17] Kojima S, Ohshima Y, Nakatsukasa H, Tsukimoto M (2017). Role of ATP as a key signaling molecule mediating radiation-induced biological effects. Dose Response.

[18] Wilhelm K, Ganesan J, Muller T, Durr C, Grimm M, Beilhack A (2010). Graft-versus-host disease is enhanced by extracellular ATP activating P2X7R. Nat Med.

[19] Bartlett R, Stokes L, Sluyter R (2014). The P2X7 receptor channel: recent developments and the use of P2X7 antagonists in models of disease. Pharmacol Rev.

[20] Surprenant A, Rassendren F, Kawashima E, North RA, Buell G (1996). The cytolytic P2Z receptor for extracellular ATP identified as a P2X receptor (P2X7). Science.

[21] De Marchi E, Pegoraro A, Adinolfi E (2021). P2X7 receptor in hematological malignancies. Front Cell Dev Biol.

[22] Filippin KJ, de Souza KFS, de Araujo Junior RT, Torquato HFV, Dias DA, Parisotto EB (2020). Involvement of P2 receptors in hematopoiesis and hematopoietic disorders, and as pharmacological targets. Purinergic Signal.

[23] Barbosa CM, Leon CM, Nogueira-Pedro A, Wasinsk F, Araujo RC, Miranda A (2011). Differentiation of hematopoietic stem cell and myeloid populations by ATP is modulated by cytokines. Cell Death Dis.

[24] Casati A, Frascoli M, Traggiai E, Proietti M, Schenk U, Grassi F (2011). Cell-autonomous regulation of hematopoietic stem cell cycling activity by ATP. Cell Death Differ.

[25] Feng W, Yang F, Wang R, Yang X, Wang L, Chen C (2016). High level P2X7-mediated signaling impairs function of hematopoietic stem/progenitor cells. Stem Cell Rev.

[26] Koldej R, Collins J, Ritchie D (2018). P2X7 polymorphisms and stem cell mobilisation. Leukemia.

[27] Pinto do OP, Kolterud A, Carlsson L (1998). Expression of the LIM-homeobox gene LH2 generates immortalized steel factor-dependent multipotent hematopoietic precursors. EMBO J.

[28] Belle JI, Petrov JC, Langlais D, Robert F, Cencic R, Shen S (2016). Repression of p53-target gene Bbc3/PUMA by MYSM1 is essential for the survival of hematopoietic multipotent progenitors and contributes to stem cell maintenance. Cell Death Differ.

[29] Tonelli C, Amati B, Morelli MJ (2016). p53 transcriptional programs in B cells upon exposure to genotoxic stress in vivo: computational analysis of next-generation sequencing data. Genom Data.

[30] Tonelli C, Morelli MJ, Bianchi S, Rotta L, Capra T, Sabo A (2015). Genome-wide analysis of p53 transcriptional programs in B cells upon exposure to genotoxic stress in vivo. Oncotarget.

[31] Purbey PK, Scumpia PO, Kim PJ, Tong AJ, Iwamoto KS, McBride WH (2017). Defined sensing mechanisms and signaling pathways contribute to the global inflammatory gene expression output elicited by ionizing radiation. Immunity.

[32] Belle JI, Wang H, Fiore A, Petrov JC, Lin YH, Feng CH, (2020). MYSM1 maintains ribosomal protein gene expression in hematopoietic stem cells to prevent hematopoietic dysfunction. JCI Insight.

[33] Snellenberg S, Cillessen SA, Van Criekinge W, Bosch L, Meijer CJ, Snijders PJ (2014). Methylation-mediated repression of PRDM14 contributes to apoptosis evasion in HPV-positive cancers. Carcinogenesis.

[34] Belkahla S, Haq Khan AU, Gitenay D, Alexia C, Gondeau C, Vo DN (2018). Changes in metabolism affect expression of ABC transporters through ERK5 and depending on p53 status. Oncotarget.

[35] Li H, Zhang Y, Strose A, Tedesco D, Gurova K, Selivanova G (2014). Integrated high-throughput analysis identifies Sp1 as a crucial determinant of p53-mediated apoptosis. Cell Death Differ.

[36] Thornborrow EC, Manfredi JJ (2001). The tumor suppressor protein p53 requires a cofactor to activate transcriptionally the human BAX promoter. J Biol Chem.

[37] McLean CY, Bristor D, Hiller M, Clarke SL, Schaar BT, Lowe CB (2010). GREAT improves functional interpretation of cis-regulatory regions. Nat Biotechnol.

[38] Kanehisa M, Goto S (2000). KEGG: kyoto encyclopedia of genes and genomes. Nucleic Acids Res.

[39] Griffith M, Griffith OL, Coffman AC, Weible JV, McMichael JF, Spies NC (2013). DGIdb: mining the druggable genome. Nat Methods.

[40] Solle M, Labasi J, Perregaux DG, Stam E, Petrushova N, Koller BH (2001). Altered cytokine production in mice lacking P2X(7) receptors. J Biol Chem.

[41] Patterson AM, Liu L, Sampson CH, Plett PA, Li H, Singh P (2020). A single radioprotective dose of prostaglandin E2 blocks irradiation-induced apoptotic signaling and early cycling of hematopoietic stem cells. Stem Cell Rep.

[42] Pietras EM (2017). Inflammation: a key regulator of hematopoietic stem cell fate in health and disease. Blood.

[43] Essers MA, Offner S, Blanco-Bose WE, Waibler Z, Kalinke U, Duchosal MA (2009). IFNalpha activates dormant haematopoietic stem cells in vivo. Nature.

[44] Sato T, Onai N, Yoshihara H, Arai F, Suda T, Ohteki T (2009). Interferon regulatory factor-2 protects quiescent hematopoietic stem cells from type I interferon-dependent exhaustion. Nat Med.

[45] Yu Q, Katlinskaya YV, Carbone CJ, Zhao B, Katlinski KV, Zheng H (2015). DNA-damage-induced type I interferon promotes senescence and inhibits stem cell function. Cell Rep.

[46] Stengel A, Schnittger S, Weissmann S, Kuznia S, Kern W, Kohlmann A (2014). TP53 mutations occur in 15.7% of ALL and are associated with MYC-rearrangement, low hypodiploidy, and a poor prognosis. Blood.

[47] Chen S, Liu Y (2019). p53 involvement in clonal hematopoiesis of indeterminate potential. Curr Opin Hematol.

[48] Weichselbaum RR, Liang H, Deng L, Fu YX (2017). Radiotherapy and immunotherapy: a beneficial liaison?. Nat Rev Clin Oncol.

[49] Deng L, Liang H, Xu M, Yang X, Burnette B, Arina A (2014). STING-dependent cytosolic DNA sensing promotes radiation-induced type I interferon-dependent antitumor immunity in immunogenic tumors. Immunity.

[50] Rodriguez-Ruiz ME, Vitale I, Harrington KJ, Melero I, Galluzzi L (2020). Immunological impact of cell death signaling driven by radiation on the tumor microenvironment. Nat Immunol.

[51] Brosh R, Sarig R, Natan EB, Molchadsky A, Madar S, Bornstein C (2010). p53-dependent transcriptional regulation of EDA2R and its involvement in chemotherapy-induced hair loss. FEBS Lett.

[52] Kwack MH, Kim JC, Kim MK (2019). Ectodysplasin-A2 induces apoptosis in cultured human hair follicle cells and promotes regression of hair follicles in mice. Biochem Biophys Res Commun.

[53] Tanikawa C, Furukawa Y, Yoshida N, Arakawa H, Nakamura Y, Matsuda K (2009). XEDAR as a putative colorectal tumor suppressor that mediates p53-regulated anoikis pathway. Oncogene.

[54] Tanikawa C, Ri C, Kumar V, Nakamura Y, Matsuda K (2010). Crosstalk of EDA-A2/XEDAR in the p53 signaling pathway. Mol Cancer Res.

[55] Wong KK, Izaguirre DI, Kwan SY, King ER, Deavers MT, Sood AK (2013). Poor survival with wild-type TP53 ovarian cancer?. Gynecol Oncol.

[56] Zeron-Medina J, Wang X, Repapi E, Campbell MR, Su D, Castro-Giner F (2013). A polymorphic p53 response element in KIT ligand influences cancer risk and has undergone natural selection. Cell.

[57] Pant V, Xiong S, Chau G, Tsai K, Shetty G, Lozano G (2016). Distinct downstream targets manifest p53-dependent pathologies in mice. Oncogene.

[58] Bilodeau MS, Arguin G, Gendron FP (2015). C/EBPbeta regulates P2X7 receptor expression in response to glucose challenge in intestinal epithelial cells. Biochem Cell Biol.

[59] Garcia-Huerta P, Diaz-Hernandez M, Delicado EG, Pimentel-Santillana M, Miras-Portugal MT, Gomez-Villafuertes R (2012). The specificity protein factor Sp1 mediates transcriptional regulation of P2X7 receptors in the nervous system. J Biol Chem.

[60] Yang R, Yu T, Kou X, Gao X, Chen C, Liu D (2018). Tet1 and Tet2 maintain mesenchymal stem cell homeostasis via demethylation of the P2rX7 promoter. Nat Commun.

[61] Sluyter R (2015). P2X and P2Y receptor signaling in red blood cells. Front Mol Biosci.

[62] Di Virgilio F, Dal Ben D, Sarti AC, Giuliani AL, Falzoni S (2017). The P2X7 receptor in infection and inflammation. Immunity.

[63] Wei J, Wang H, Wang H, Wang B, Meng L, Xin Y (2019). The role of NLRP3 inflammasome activation in radiation damage. Biomed Pharmacother.

[64] Ratajczak MZ, Bujko K, Cymer M, Thapa A, Adamiak M, Ratajczak J (2020). The Nlrp3 inflammasome as a “rising star” in studies of normal and malignant hematopoiesis. Leukemia.

[65] Keystone EC, Wang MM, Layton M, Hollis S, McInnes IB, Team DCS (2012). Clinical evaluation of the efficacy of the P2X7 purinergic receptor antagonist AZD9056 on the signs and symptoms of rheumatoid arthritis in patients with active disease despite treatment with methotrexate or sulphasalazine. Ann Rheum Dis.

[66] Eser A, Colombel JF, Rutgeerts P, Vermeire S, Vogelsang H, Braddock M (2015). Safety and efficacy of an oral inhibitor of the purinergic receptor P2X7 in adult patients with moderately to severely active Crohn’s disease: a randomized placebo-controlled, double-blind, phase IIa study. Inflamm Bowel Dis.

[67] Colarusso C, Terlizzi M, Molino A, Pinto A, Sorrentino R (2017). Role of the inflammasome in chronic obstructive pulmonary disease (COPD). Oncotarget.

[68] He X, Wan J, Yang X, Zhang X, Huang D, Li X (2021). Bone marrow niche ATP levels determine leukemia-initiating cell activity via P2X7 in leukemic models. J Clin Invest.

[69] Pellagatti A, Marafioti T, Paterson JC, Barlow JL, Drynan LF, Giagounidis A (2010). Induction of p53 and up-regulation of the p53 pathway in the human 5q- syndrome. Blood.

[70] Dutt S, Narla A, Lin K, Mullally A, Abayasekara N, Megerdichian C (2011). Haploinsufficiency for ribosomal protein genes causes selective activation of p53 in human erythroid progenitor cells. Blood.

[71] Ceccaldi R, Parmar K, Mouly E, Delord M, Kim JM, Regairaz M (2012). Bone marrow failure in Fanconi anemia is triggered by an exacerbated p53/p21 DNA damage response that impairs hematopoietic stem and progenitor cells. Cell Stem Cell.

[72] Wilson NK, Schoenfelder S, Hannah R, Sanchez Castillo M, Schutte J, Ladopoulos V (2016). Integrated genome-scale analysis of the transcriptional regulatory landscape in a blood stem/progenitor cell model. Blood.

[73] Bolger AM, Lohse M, Usadel B (2014). Trimmomatic: a flexible trimmer for Illumina sequence data. Bioinformatics.

[74] Kim D, Pertea G, Trapnell C, Pimentel H, Kelley R, Salzberg SL (2013). TopHat2: accurate alignment of transcriptomes in the presence of insertions, deletions and gene fusions. Genome Biol.

[75] Langmead B, Trapnell C, Pop M, Salzberg SL (2009). Ultrafast and memory-efficient alignment of short DNA sequences to the human genome. Genome Biol.

[76] Trapnell C, Pachter L, Salzberg SL (2009). TopHat: discovering splice junctions with RNA-Seq. Bioinformatics.

[77] Liao Y, Smyth GK, Shi W (2014). featureCounts: an efficient general purpose program for assigning sequence reads to genomic features. Bioinformatics.

[78] Bolstad B. preprocessCore: a collection of pre-processing functions. R package version 1.44.0. 2018. https://github.com/bmbolstad/preprocessCore.

[79] Mevik BH, Wehrens R (2007). The pls package: principal component and partial least squares regression in R. J Stat Softw.

[80] Robinson MD, Oshlack A (2010). A scaling normalization method for differential expression analysis of RNA-seq data. Genome Biol.

[81] Thorvaldsdottir H, Robinson JT, Mesirov JP (2013). Integrative Genomics Viewer (IGV): high-performance genomics data visualization and exploration. Brief Bioinform.

[82] Huang DW, Sherman BT, Tan Q, Collins JR, Alvord WG, Roayaei J (2007). The DAVID Gene Functional Classification Tool: a novel biological module-centric algorithm to functionally analyze large gene lists. Genome Biol.

[83] Subramanian A, Tamayo P, Mootha VK, Mukherjee S, Ebert BL, Gillette MA (2005). Gene set enrichment analysis: a knowledge-based approach for interpreting genome-wide expression profiles. Proc Natl Acad Sci USA.

[84] Zhang Y, Liu T, Meyer CA, Eeckhoute J, Johnson DS, Bernstein BE (2008). Model-based analysis of ChIP-Seq (MACS). Genome Biol.

[85] Heinz S, Benner C, Spann N, Bertolino E, Lin YC, Laslo P (2010). Simple combinations of lineage-determining transcription factors prime cis-regulatory elements required for macrophage and B cell identities. Mol Cell.

