# Supplementary material for: p53-dependent induction of P2X7 on hematopoietic stem and progenitor cells regulates hematopoietic response to genotoxic stress
Source: Cell Death Dis. 2021 Oct 8;12(10):923. doi: 10.1038/s41419-021-04202-9 (PMC8501024; doi:10.1038/s41419-021-04202-9)
Supplement: Supplementary file 1 — Supplemental Materials [file 41419_2021_4202_MOESM1_ESM.pdf]

SUPPLEMENTAL MATERIALS

p53-Dependent Induction of P2X7 on Hematopoietic Stem and Progenitor Cells Regulates Hematopoietic Response to Genotoxic Stress

Lin Tze Tung[1,2] #, HanChen Wang[1,2,3] #, Jad I Belle[1,2], Jessica C Petrov[1,2], David Langlais[2,3,4,5], Anastasia Nijnik[1,2,*]

[1] Department of Physiology, McGill University, Montreal, QC, Canada

[2] McGill University Research Centre on Complex Traits, McGill University, QC, Canada

[3] Department of Human Genetics, McGill University, Montreal, QC, Canada

[4] McGill University Genome Centre, McGill University, Montreal, QC, Canada

[5] Department of Microbiology and Immunology, McGill University, Montreal, QC, Canada

# These authors contributed equally to this work and are listed in the alphabetical order of their surnames.

Content | **Pages**

Content	Pages
Supplemental Figures and Figure Legends S1-S11	2-23
Supplemental Data Table Legends S1-S3	24-25
Supplemental Methods Tables S4-6	26-29

SUPPLEMENTAL FIGURES

Figure S1. Consolidation of ChIP-Seq data on p53 genome-wide DNA-binding sites across different studies and cell types. (Related to Figure 2A).

Heat map showing the binding intensities of p53 or phospho-p53 at the 7,384 p53 binding sites identified in our ChIP-Seq experiments from untreated (UT) and irradiated (3-Gy, 3 hours) Ba/F3 and HPC7 hematopoietic progenitor cells. The sites are ordered as in Figure 2A, based on fold change in binding intensity following irradiation (IR) and organized into three groups: Group I is composed of peaks with ≥1.5 fold increase in p53 binding following IR; Group II is composed of peaks with minimal change in p53 binding (FC ≤ |1.5|); and Group III is composed of peaks with ≥1.5 fold decrease in p53 binding following IR. Binding intensities at ± 1kb around the p53 peak centers are plotted. The p53 ChIP-Seq from Ba/F3 and HPC7 cells represents our datasets and is described in Figure 2A. The other data is from public datasets, downloaded and re-analyzed using our pipeline. These include: p53 ChIP-Seq from splenic B and non-B cells with or without irradiation (7-Gy, 4hours, GSE71180) [29-30], phospho-p53 ChIP-Seq from bone marrow derived macrophages (BMDM) with or without irradiation (6-Gy, various time points, GSE100963) [31], and p53 ChIP-Seq from mouse embryo fibroblasts (MEFs) with or without doxorubicin (DOX) treatment (GSE46240) [5]; all datasets are from mouse.

Figure S1

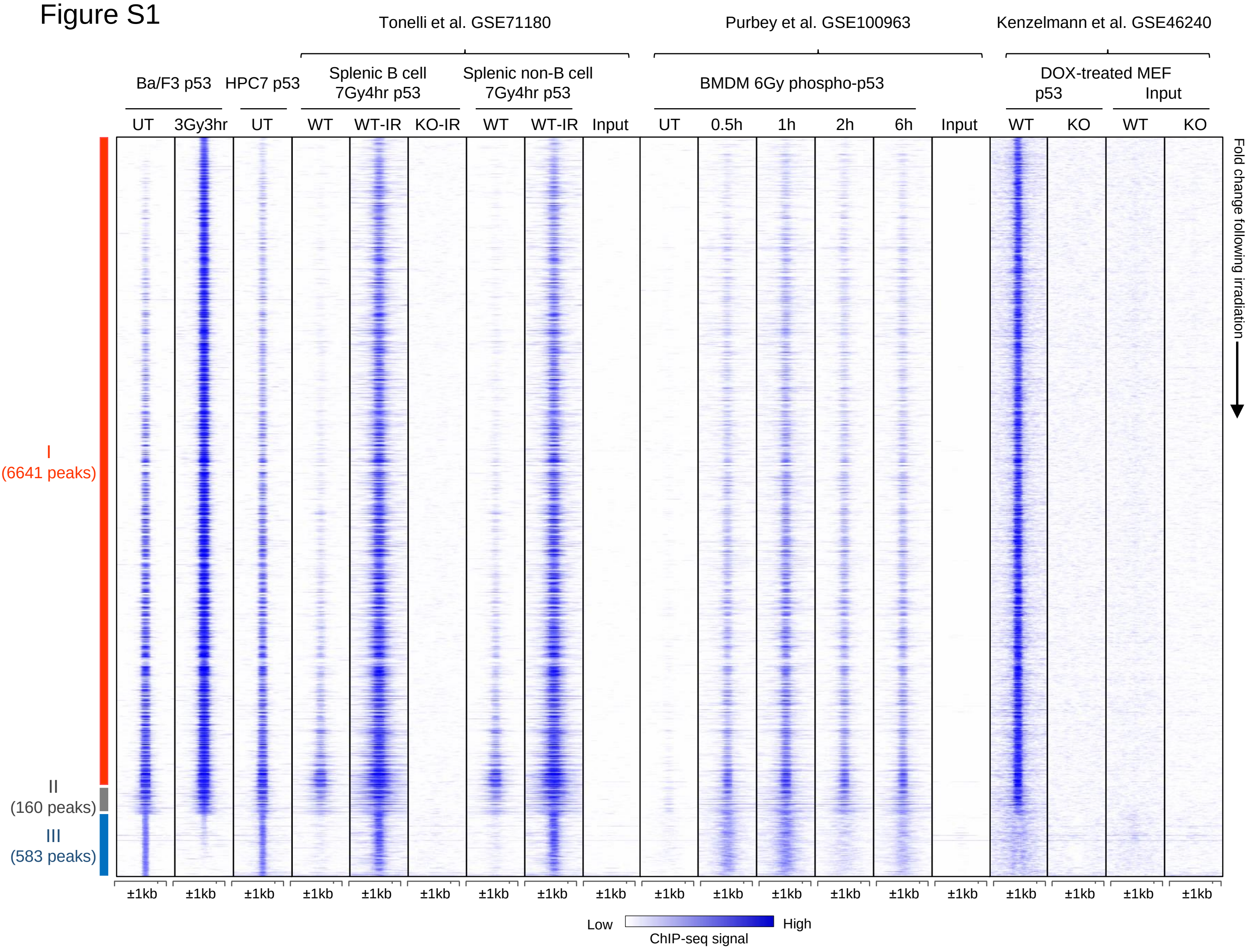
Tonelli et al. GSE71180
Purbey et al. GSE100963
Kenzelmann et al. GSE46240
Ba/F3 p53
HPC7 p53
Splenic B cell 7Gy4hr p53
Splenic non-B cell 7Gy4hr p53
BMDM 6Gy phospho-p53
DOX-treated MEF
p53
Input
UT
3Gy3hr
UT
WT
WT-IR
KO-IR
WT
WT-IR
Input
UT
0.5h
1h
2h
6h
Input
WT
KO
WT
KO
Fold change following irradiation
I (6641 peaks)
II (160 peaks)
III (583 peaks)
±1kb
Low
High
ChIP-seq signal

Figure S2. Further analyses of the p53 and H3K27ac ChIP-Seq data from hematopoietic progenitor cells. (Related to Figure 2A).

(A-B) Histograms display changes in p53 **(A)** and histone H3K27ac **(B)** ChIP-Seq binding intensities around Group I-III p53 DNA-binding peaks in Ba/F3 hematopoietic progenitor cells with and without irradiation (3 hours, 3 Gy). **(C)** Results of the *de novo* motif finding analyses at Group I-III p53 DNA-binding peaks; top 2 motifs for each p53 peak group are displayed. The analysis searched for over-represented motifs within ± 100 bp to the p53 peak centers. **(D)** Pipeline used to identify novel p53-target genes within our RNA/ChIP-seq datasets. We screened our list of 131 p53 target genes against the list of known p53 signaling pathway genes identified by KEGG pathway mmu04115 [38], and against lists of p53-target genes from 14 previous RNA/ChIP-seq studies conducted in other cell types and reviewed by Fischer M. *et al.* [4]. Additionally, we performed an automated search of PubMed for publications that include the name of each of the putative p53-target genes from our datasets together with the term "p53" in the title or in the abstract of the article. Based on these criteria, we identified 38 potentially novel p53-target genes. We further focussed our analyses on the genes encoding "druggable" proteins, by searching for the available pharmacological agents for each gene/protein using the DGIdb database [39].

Figure S2

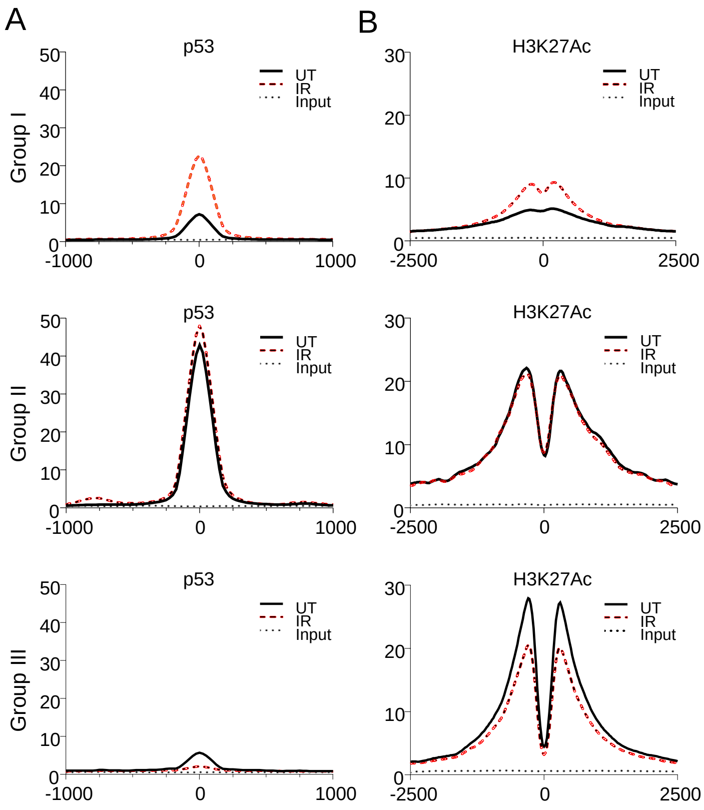

C

Group	Motif	Name	Adj. q-value	% Peaks with motif (%background with motif)
I	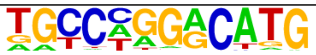	p53	10^{-4141}	59% (2.9%)
	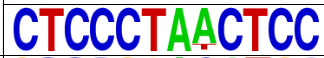	PRDM14	10^{-2084}	12% (0.01%)
II	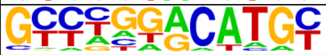	p53	10^{-64}	47% (3.4%)
	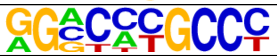	Sp1	10^{-34}	47% (9.1%)
III	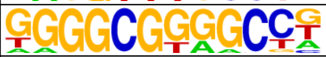	Sp1	10^{-168}	71% (20%)
	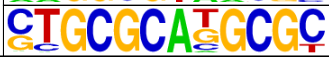	NRF1	10^{-86}	35% (6.7%)

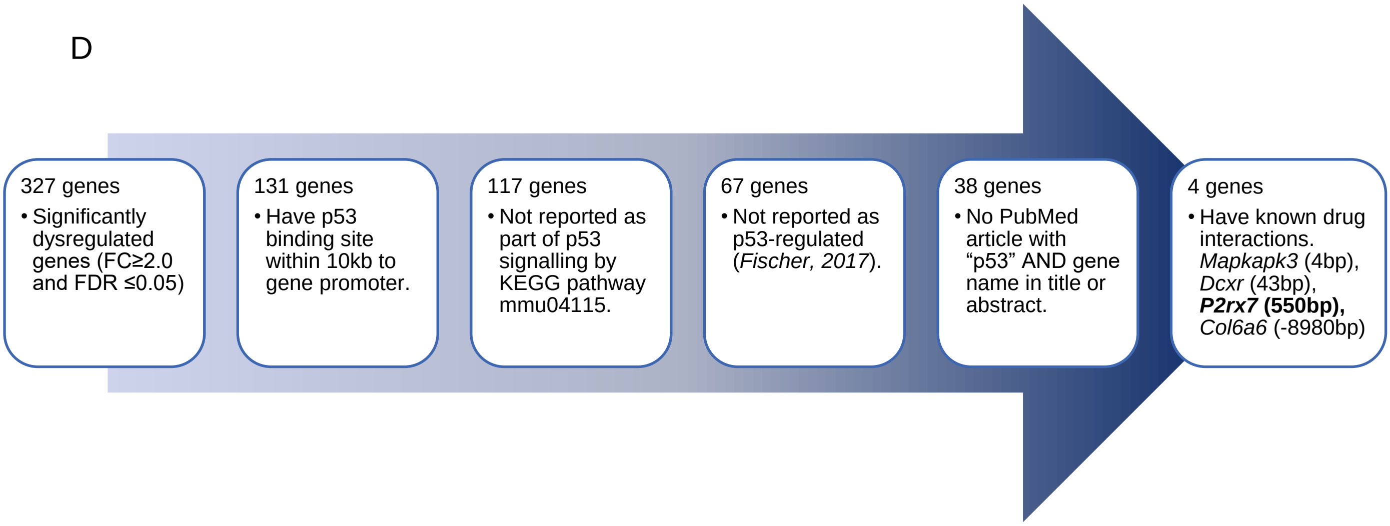

Figure S3. P2X7 expression on HSCs and hematopoietic progenitor cells. (Related to Figures 5D-E).

Mice of WT and p53KO genotypes were analyzed by flow cytometry, with and without whole body irradiation (3 Gy, 6 hours), using P2X7-KO mouse as a negative control. **(A)** Representative flow cytometry histograms of P2X7 expression on long term HSCs (LT-HSCs, gated as $Lin^{-}cKit^{+}Sca1^{+}CD150^{+}CD48^{-}CD34^{-}Flt3^{-}$); pre-MkE progenitors (gated as $Lin^{-}cKit^{+}Sca1^{-}CD41^{-}CD16/32^{-}CD150^{+}CD105^{-}$); and CFU-E progenitors (gated as $Lin^{-}cKit^{+}Sca1^{-}CD41^{-}CD16/32^{-}CD150^{-}CD105^{+}$). **(B)** Quantification of P2X7 expression, with the mean fluorescence intensity of P2X7 staining plotted for each cell population. Data is from 3-4 mice per genotype per condition, consolidated from 2-3 independent experiments. Bars represent means and standard errors on the mean (SEM); statistical analysis by ANOVA followed by Sidak's multiple comparison post-hoc test to compare WT versus WT-IR and p53KO versus p53KO-IR datasets; * $p<0.05$, ** $p<0.01$, or ns - not significant. Cells are gated as: $Lin^{-}cKit^{+}Sca1^{-}$ followed by $CD34^{+}CD16/32^{-}$ for the common myeloid progenitor (CMP), $CD34^{+}CD16/32^{+}$ for the granulocyte monocyte progenitor (GMP), and $CD41^{+}CD150^{+}CD16/32^{-}$ for the megakaryocyte progenitor (MkP). Complementary data for other HSPC cell populations is presented in Figure 5D-E.

Figure S3

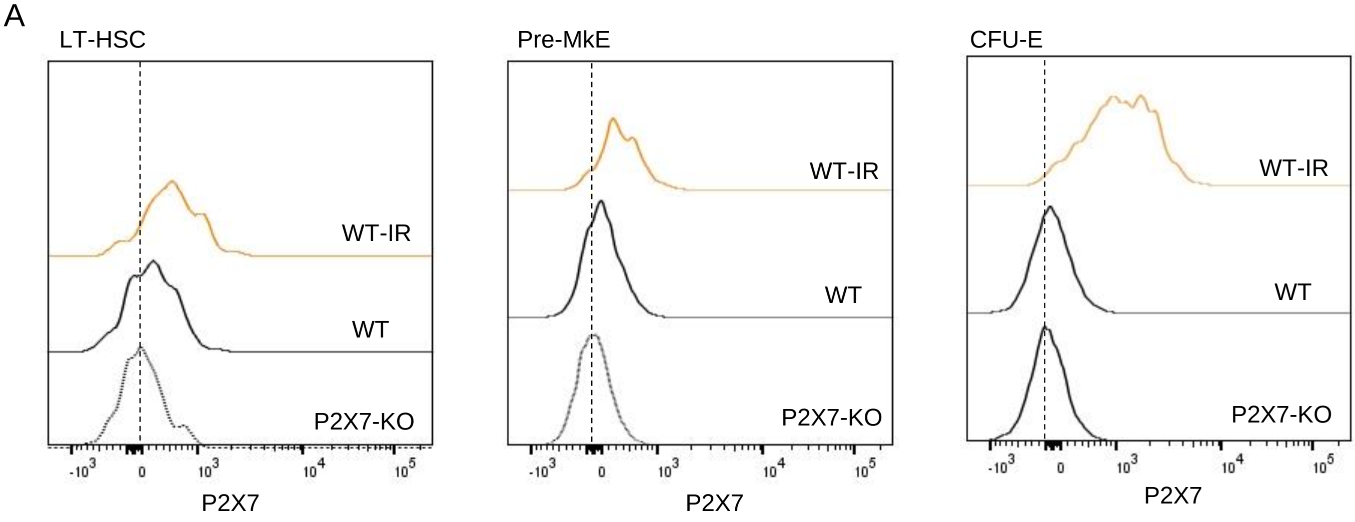
A
LT-HSC
WT-IR
WT
P2X7-KO
P2X7
Pre-MkE
WT-IR
WT
P2X7-KO
P2X7
CFU-E
WT-IR
WT
P2X7-KO
P2X7

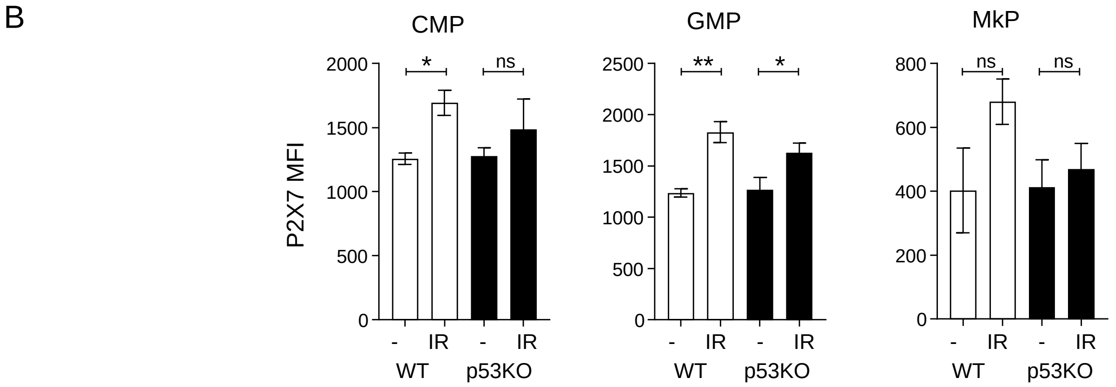
B
CMP
GMP
MkP
P2X7 MFI
*
ns
**
*
ns
ns
WT
p53KO
IR

Figure S4. Analysis of hematopoietic stem and progenitor cell numbers in the bone marrow of P2X7-knockout (KO) and wild type control (WT) mice. Data is from two independent experiments with 3-4 mice analyzed per genotype per experiment. Samples were analyzed by flow cytometry, and each cell population is presented as a percentage of live bone marrow cells and as an absolute cell number per one tibia and femur. Bars represent means and standard errors on the mean (SEM); statistical analyses by Student's *t*-test, * $p<0.05$, ** $p<0.01$, or not significant if not indicated. **(A-C)** Analysis of HSC, multipotent progenitor (MPP), and lineage primed progenitor cells, with quantification presented in bar charts in (A-B) and gating strategies and representative flow cytometry plots in (C), showing the average cell frequency within each gate as mean ± SD. Cells are gated as: $Lin^{-}cKit^{+}Sca1^{+}$ for LSK, followed by $CD150^{+}CD48^{-}CD34^{-}Flt3^{-}$ for LT-HSCs, $CD150^{+}CD48^{-}CD34^{+}Flt3^{-}$ for MPP1, $CD150^{+}CD48^{+}CD34^{+}Flt3^{-}$ for MPP2, $CD150^{-}CD48^{+}CD34^{+}Flt3^{-}$ for MPP3, and $CD150^{-}CD48^{+}CD34^{+}Flt3^{+}$ for MPP4, or as $Lin^{-}cKit^{+}Sca1^{-}$ for LK, followed by $CD34^{-}CD16/32^{-}$ for megakaryocyte erythroid progenitor (MEP), $CD34^{+}CD16/32^{-}$ for common myeloid progenitor (CMP), and $CD34^{+}CD16/32^{+}$ for granulocyte monocyte progenitor (GMP), and $Lin^{-}IL7R\alpha^{+}cKit^{lo}Sca1^{lo}$ for common lymphoid progenitor (CLP). **(D-E)** Further characterization of erythroid and megakaryocyte progenitors; cells are gated as LK followed by $CD41^{-}CD16/32^{-}CD150^{+}CD105^{-}$ for pre-megakaryocyte-erythroid progenitor (pre-MkE), $CD41^{-}CD16/32^{-}CD150^{+}CD105^{+}$ for pre-CFU-E erythroid progenitor, $CD41^{-}CD16/32^{-}CD150^{-}CD105^{+}$ for CFU-E erythroid progenitor, and $CD150^{+}CD41^{+}$ for megakaryocyte progenitor (MkP).

Figure S4

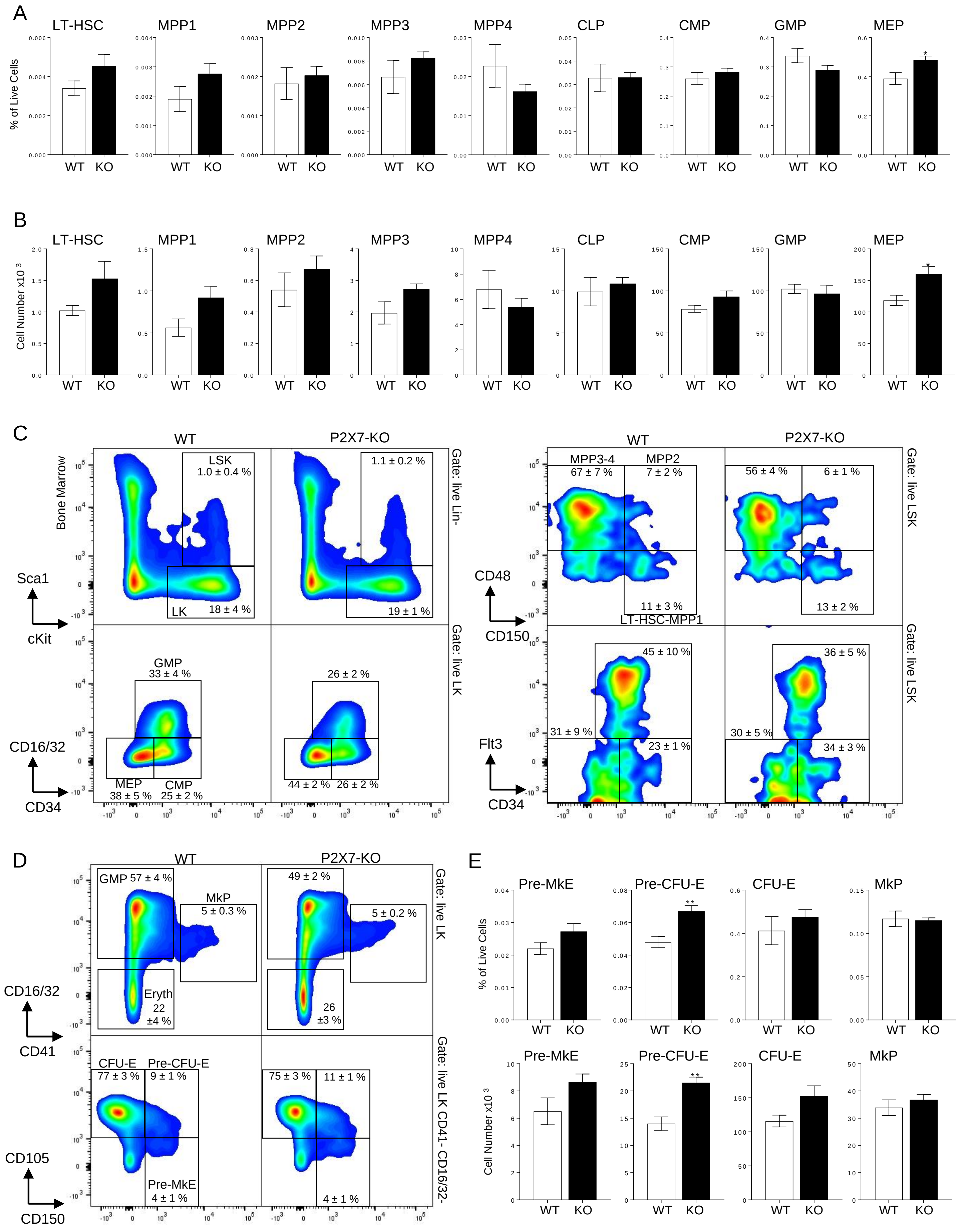
A
LT-HSC
MPP1
MPP2
MPP3
MPP4
CLP
CMP
GMP
MEP
% of Live Cells
WT KO
B
Cell Number x10 3
C
WT
P2X7-KO
Bone Marrow
LSK
1.0 ± 0.4 %
1.1 ± 0.2 %
LK
18 ± 4 %
19 ± 1 %
Sca1
cKit
Gate: live Lin-
GMP
33 ± 4 %
26 ± 2 %
MEP
38 ± 5 %
CMP
25 ± 2 %
44 ± 2 %
26 ± 2 %
CD16/32
CD34
Gate: live LK
MPP3-4
67 ± 7 %
MPP2
7 ± 2 %
56 ± 4 %
6 ± 1 %
11 ± 3 %
13 ± 2 %
LT-HSC-MPP1
CD48
CD150
Gate: live LSK
45 ± 10 %
36 ± 5 %
31 ± 9 %
30 ± 5 %
23 ± 1 %
34 ± 3 %
Flt3
D
GMP 57 ± 4 %
49 ± 2 %
MkP
5 ± 0.3 %
5 ± 0.2 %
Eryth
22
±4 %
26
±3 %
CD41
CFU-E
Pre-CFU-E
77 ± 3 %
9 ± 1 %
75 ± 3 %
11 ± 1 %
Pre-MkE
4 ± 1 %
4 ± 1 %
CD105
Gate: live LK CD41- CD16/32-
E
Pre-MkE
Pre-CFU-E
CFU-E
MkP

Figure S5. Analysis of B cell lineage development in the bone marrow of P2X7-knockout (KO) and wild type control (WT) mice. Data is from two independent experiments with 3-4 mice analyzed per genotype per experiment. **(A-B)** Representative flow cytometry plots and gating strategies, with the average cell frequency in each gate presented as mean ± SD. Cells are gated as $B220^{+}$ for the total B cell linage cells, and $IgM^{-}CD19^{-}CD43^{+}$ for pre-pro-B cells, $IgM^{-}CD19^{+}CD43^{+}$ for pro-B cells, $IgM^{-}CD19^{+}CD43^{-}$ for pre-B cells, $IgM^{+}IgD^{-}$ for immature B cells, and $IgM^{+}IgD^{+}$ for mature B cells. **(C-F)** Cell quantification presented in bar charts, with each cell population shown as a percentage of live bone marrow cells and as an absolute cell number per one tibia and femur. Bars represent means ± SEM; statistical analysis by Student's *t*-test, * $p<0.05$, ** $p<0.01$, *** $p<0.001$ or not significant if not indicated.

Figure S5

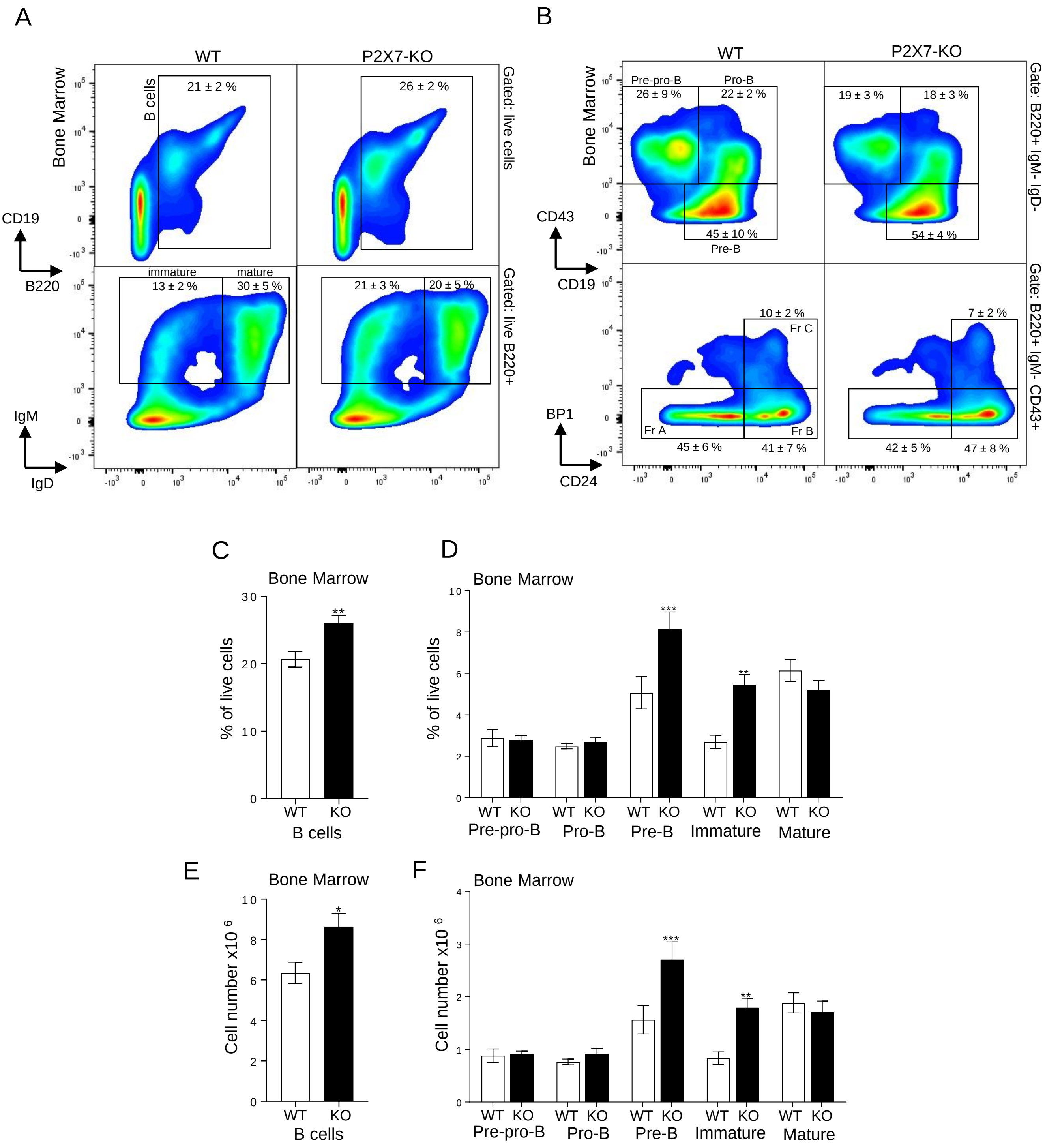
A
WT
P2X7-KO
Bone Marrow
B cells
21 ± 2 %
26 ± 2 %
Gated: live cells
CD19
B220
immature
mature
13 ± 2 %
30 ± 5 %
21 ± 3 %
20 ± 5 %
Gated: live B220+
IgM
IgD
B
WT
P2X7-KO
Bone Marrow
Pre-pro-B
Pro-B
26 ± 9 %
22 ± 2 %
19 ± 3 %
18 ± 3 %
45 ± 10 %
Pre-B
54 ± 4 %
Gate: B220+ IgM- IgD-
CD43
CD19
10 ± 2 %
Fr C
7 ± 2 %
Fr A
Fr B
45 ± 6 %
41 ± 7 %
42 ± 5 %
47 ± 8 %
Gate: B220+ IgM- CD43+
BP1
CD24
C
Bone Marrow
% of live cells
WT
KO
B cells
D
Bone Marrow
% of live cells
Pre-pro-B
Pro-B
Pre-B
Immature
Mature
E
Bone Marrow
Cell number x10 6
B cells
F
Bone Marrow
Cell number x10 6
Pre-pro-B
Pro-B
Pre-B
Immature
Mature

Figure S6. B cell characterization in the spleen of P2X7-knockout (KO) and wild type control (WT) mice. Data is from one experiment with 4-5 mice analyzed per genotype. **(A-D)** Cell quantification presented in bar charts, with each cell population shown as a percentage of live splenocytes and as an absolute cell number per spleen organ. Bars represent means ± SEM; statistical analysis by Student's *t*-test, not significant if not indicated. **(E)** Representative flow cytometry plots and gating strategies, with the average cell frequency in each gate presented as mean ± SD. Cells are gated as $B220^{+}$ for the total B cell linage cells, and $CD21^{-}CD23^{-}$ for transitional B cells, $CD21^{+}CD23^{+}$ for follicular B cells, and $CD21^{hi}CD23^{lo}$ for marginal zone B cells.

Figure S6

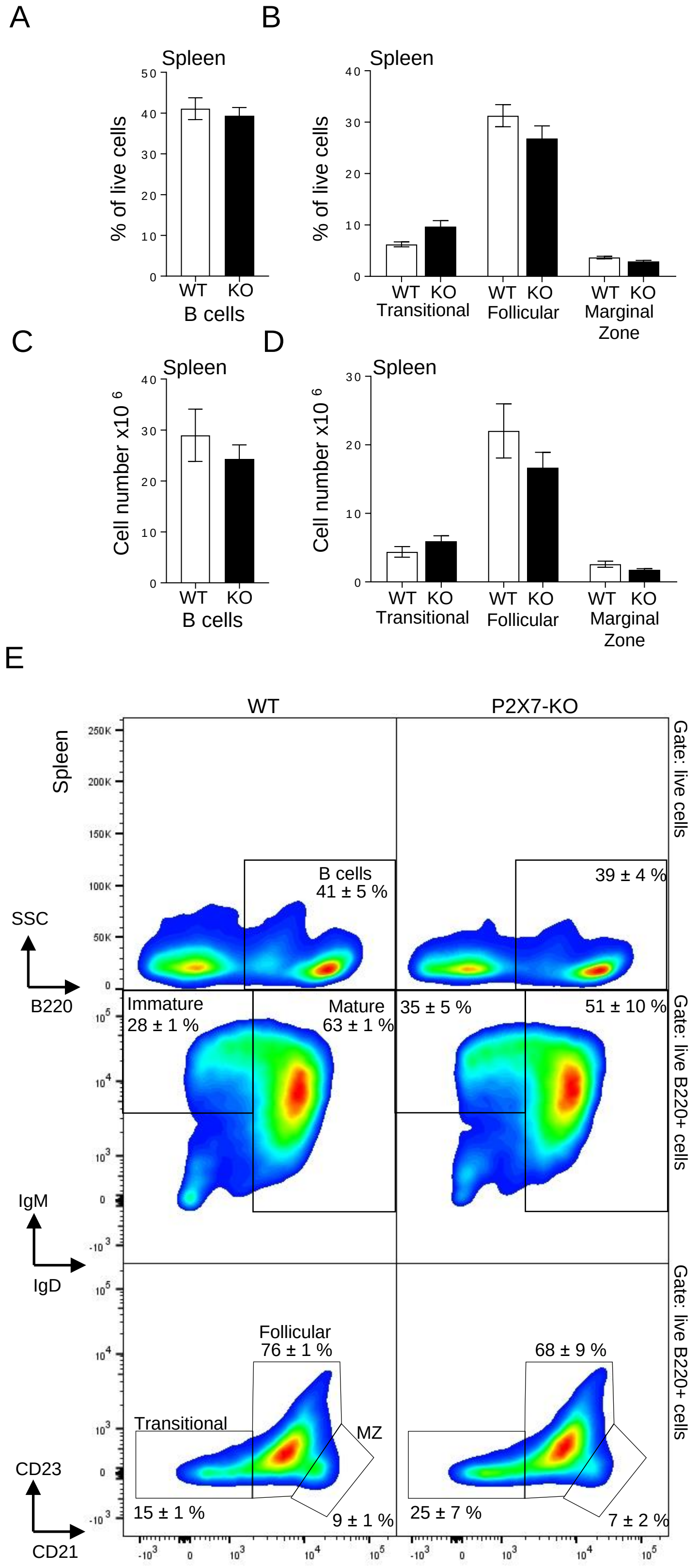
A
B
Spleen
% of live cells
WT KO
B cells
Spleen
% of live cells
WT KO
Transitional
WT KO
Follicular
WT KO
Marginal Zone
C
D
Spleen
Cell number x10 6
WT KO
B cells
Spleen
Cell number x10 6
WT KO
Transitional
WT KO
Follicular
WT KO
Marginal Zone
E
WT
P2X7-KO
Spleen
SSC
B220
B cells
41 ± 5 %
39 ± 4 %
Gate: live cells
IgM
IgD
Immature
28 ± 1 %
Mature
63 ± 1 %
35 ± 5 %
51 ± 10 %
Gate: live B220+ cells
CD23
CD21
Follicular
76 ± 1 %
Transitional
MZ
15 ± 1 %
9 ± 1 %
68 ± 9 %
25 ± 7 %
7 ± 2 %
Gate: live B220+ cells

Figure S7. Characterization of thymic T cell development and splenic T cell populations in P2X7-knockout (KO) and wild type control (WT) mice. Data is from one experiment with 4-5 mice analyzed per genotype. **(A-D)** Cell quantification presented in bar charts, with each cell population shown as a percentage of live thymocytes or splenocytes, and as an absolute cell number per thymus or spleen organ. Bars represent means ± SEM; statistical analyses by Student's *t*-test, not significant if not indicated. **(E-F)** Representative flow cytometry plots and gating strategies, analyzing thymic T cell development and T cell frequencies and activation states in the spleen; the average cell frequency in each gate is presented as mean ± SD. Cells are gated as $CD4^-CD8^-$ for double negative (DN) thymocytes, $CD4^+CD8^+$ for double positive (DP) thymocytes, $CD4^+CD8^-$ and $CD4^-CD8^+$ for single-positive thymocytes; $CD3^+CD4^+CD8^-$ and $CD3^+CD4^-CD8^+$ for the helper and cytotoxic T cells in the spleen; and $CD3^-$ $NK1.1^+$ for splenic NK cells; CD44 and CD62L markers are used to analyze the activation status of T cells.

Figure S7

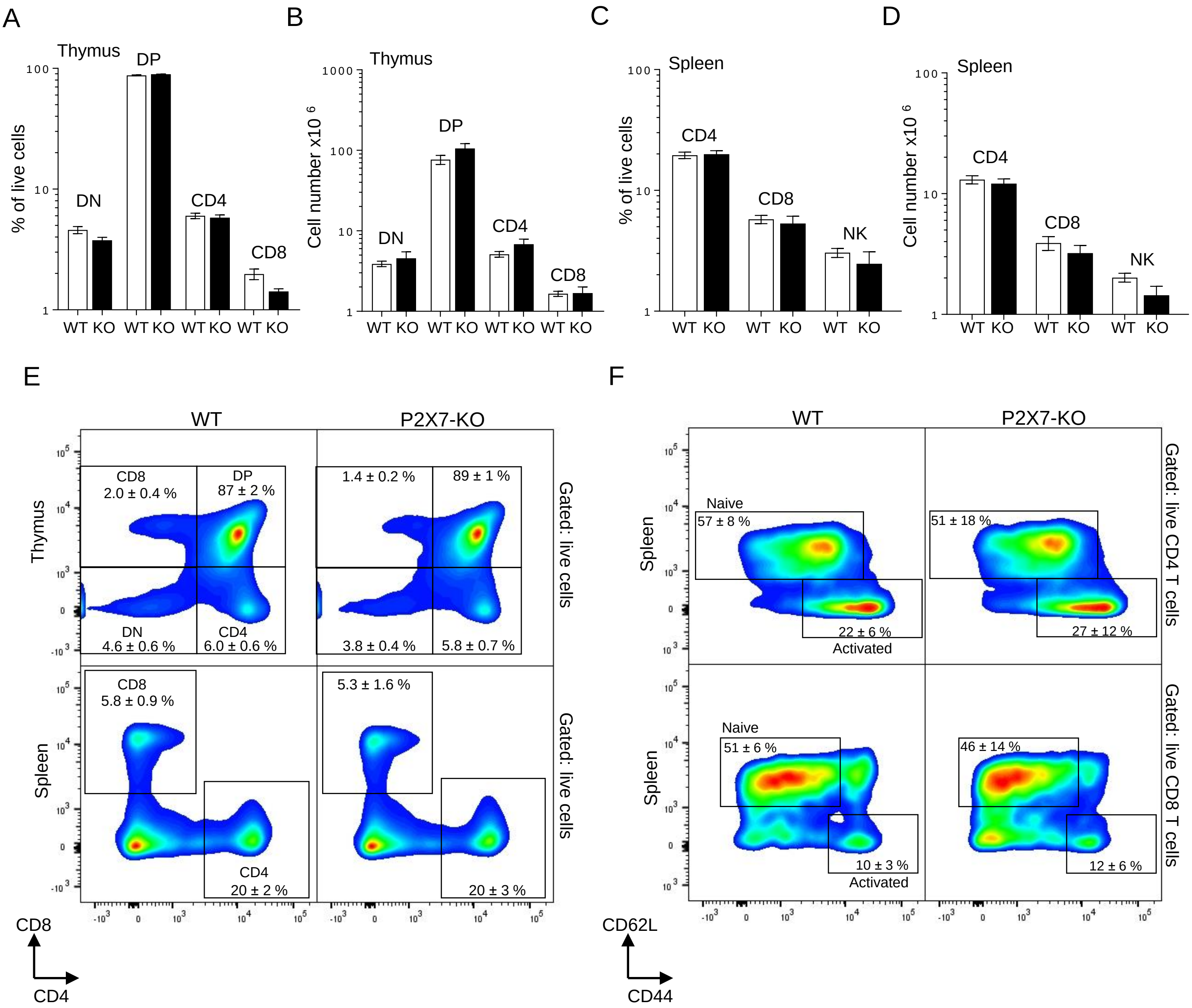

A
Thymus
% of live cells
DN
DP
CD4
CD8
WT KO WT KO WT KO WT KO
B
Thymus
Cell number x10 6
DN
DP
CD4
CD8
WT KO WT KO WT KO WT KO
C
Spleen
% of live cells
CD4
CD8
NK
WT KO WT KO WT KO
D
Spleen
Cell number x10 6
CD4
CD8
NK
WT KO WT KO WT KO
E
WT
P2X7-KO
Thymus
CD8
2.0 ± 0.4 %
DP
87 ± 2 %
DN
4.6 ± 0.6 %
CD4
6.0 ± 0.6 %
1.4 ± 0.2 %
89 ± 1 %
3.8 ± 0.4 %
5.8 ± 0.7 %
Gated: live cells
Spleen
CD8
5.8 ± 0.9 %
CD4
20 ± 2 %
5.3 ± 1.6 %
20 ± 3 %
Gated: live cells
CD8
CD4
F
WT
P2X7-KO
Spleen
Naive
57 ± 8 %
22 ± 6 %
Activated
51 ± 18 %
27 ± 12 %
Gated: live CD4 T cells
Spleen
Naive
51 ± 6 %
10 ± 3 %
Activated
46 ± 14 %
12 ± 6 %
Gated: live CD8 T cells
CD62L
CD44

Figure S8. Characterization of myeloid cell numbers in the bone marrow and spleen of P2X7-knockout (KO) and wild type control (WT) mice. Data is from one experiments with 4-5 mice analyzed per genotype. **(A)** Cell quantification presented in bar charts, with the $CD11b^+$ myeloid cell population shown as a percentage of live cells within the tissue, and as an absolute cell number. Bars represent means ± SEM; statistical analysis by Student's *t*-test, not significant if not indicated; bone marrow quantification is per one tibia and femur. **(B)** Representative flow cytometry plots and gating; the average cell frequency in the $CD11b^+$ myeloid cell gate is presented as mean ± SD.

Figure S8

A

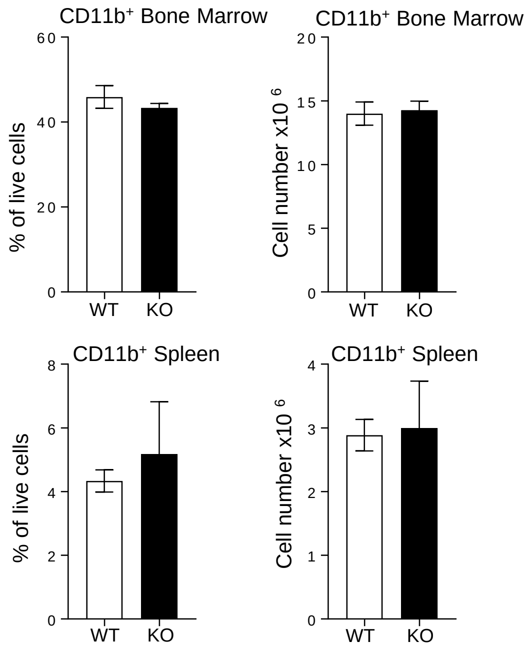

CD11b+ Bone Marrow
% of live cells
60
40
20
0
WT
KO
CD11b+ Bone Marrow
Cell number x10 6
20
15
10
5
0
WT
KO
CD11b+ Spleen
% of live cells
8
6
4
2
0
WT
KO
CD11b+ Spleen
Cell number x10 6
4
3
2
1
0
WT
KO

B

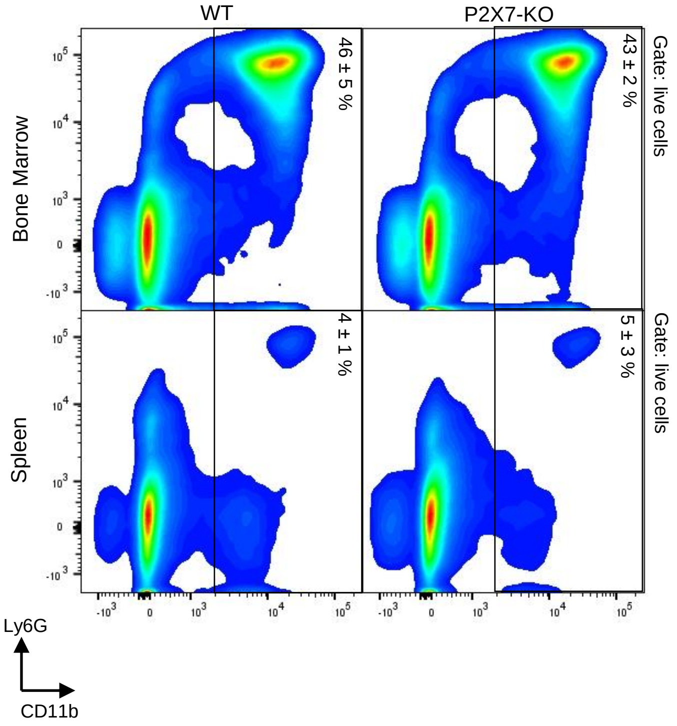

WT
P2X7-KO
Bone Marrow
Spleen
46 ± 5 %
43 ± 2 %
4 ± 1 %
5 ± 3 %
Gate: live cells
Gate: live cells
Ly6G
CD11b

Figure S9. Hematology analysis of P2X7-KO and WT mice. Data is from 5 mice per genotype; bars represent means ± SEM; statistical analysis by Student's *t*-test, not significant if not indicated. **(A)** Red blood cell (RBC), platelet, and white blood cell (WBC) counts. **(B)** Neutrophil and lymphocyte counts. **(C)** Characterization of erythroid phenotype including hemoglobin concentration, hematocrit, mean corpuscular volume (MCV), mean corpuscular hemoglobin (MCH), mean corpuscular hemoglobin concentration (MCHC), and the percentage of reticulocytes.

Figure S9

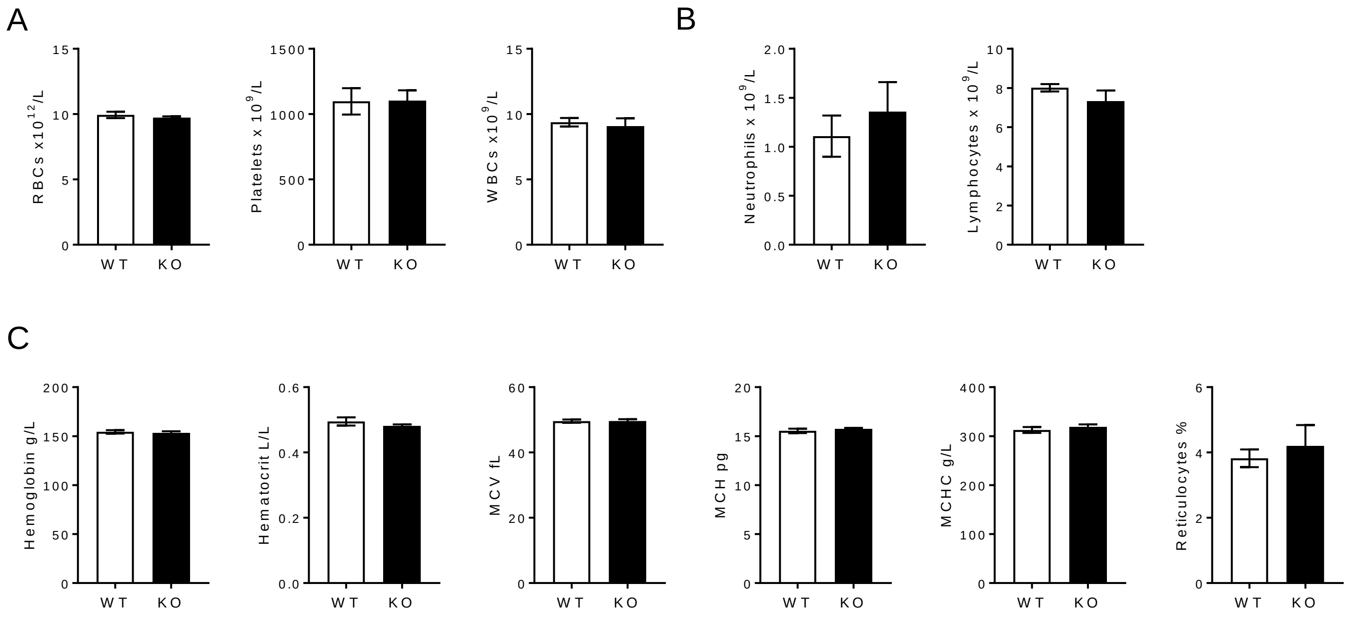
A
B
C
RBCs x10^12/L
Platelets x 10^9/L
WBCs x10^9/L
Neutrophils x 10^9/L
Lymphocytes x 10^9/L
Hemoglobin g/L
Hematocrit L/L
MCV fL
MCH pg
MCHC g/L
Reticulocytes %
WT
KO

Figure S10. Analysis of P2X7-KO hematopoietic stem cell function in a competitive bone marrow transplantation model.

Bone marrow from P2X7-KO or control WT mice was mixed with CD45.1-marked competitor bone marrow in a 1:1 ratio, and reconstituted into two independent groups of wild type B6-SJL lethality irradiated recipients. The recipients were analyzed for the relative contribution of $CD45.2^{+}$ P2X7-KO and WT donor cells to the different hematopoietic cell populations in the bone marrow and spleen. Data is from 3-5 mice per group; bars represent means and standard errors on the mean (SEM); statistical analysis by Student's *t*-test; not significant if significance is not indicated. **(A)** Schematic representation of the mouse to mouse competitive bone marrow transplantation study. **(B)** Analysis of HSCs, MPPs and **(C)** lineage primed progenitor cells. HSC-MPP cells are gated as: $Lin^{-}cKit^{+}Sca1^{+}$ followed by $CD150^{+}CD48^{-}CD34^{-}Flt3^{-}$ for LT-HSCs, $CD150^{+}CD48^{-}CD34^{+}Flt3^{-}$ for MPP1, $CD150^{+}CD48^{+}CD34^{+}Flt3^{-}$ for MPP2, $CD150^{-}CD48^{+}CD34^{+}Flt3^{-}$ for MPP3, and $CD150^{-}CD48^{+}CD34^{+}Flt3^{+}$ for MPP4. Other progenitors are gated as $Lin^{-}cKit^{+}Sca1^{-}$, followed by $CD150^{+}CD41^{+}$ for megakaryocyte progenitors (MkP), $CD34^{-}CD16/32^{-}$ for megakaryocyte erythroid progenitors (MEP), $CD34^{+}CD16/32^{-}$ for common myeloid progenitors (CMP), $CD34^{+}CD16/32^{+}$ for granulocyte monocyte progenitors (GMP), and $Lin^{-}IL7R\alpha^{+}cKit^{lo}Sca1^{lo}$ for common lymphoid progenitors (CLP). **(D-E)** Analysis of lymphoid and myeloid cells, gated as $B220^{+}$ for B cells, $CD3^{+}CD4^{+}$ and $CD3^{+}CD8^{+}$ for T cells, $CD3^{-}NK1.1^{+}$ for NK cells, $CD11b^{+}Ly6G^{-}Ly6C^{+}$ for monocytes, $CD11b^{+}Ly6C^{-}Ly6G^{+}$ for neutrophils, and $CD71^{+}$ for bone marrow erythroid precursor cells.

Figure S10

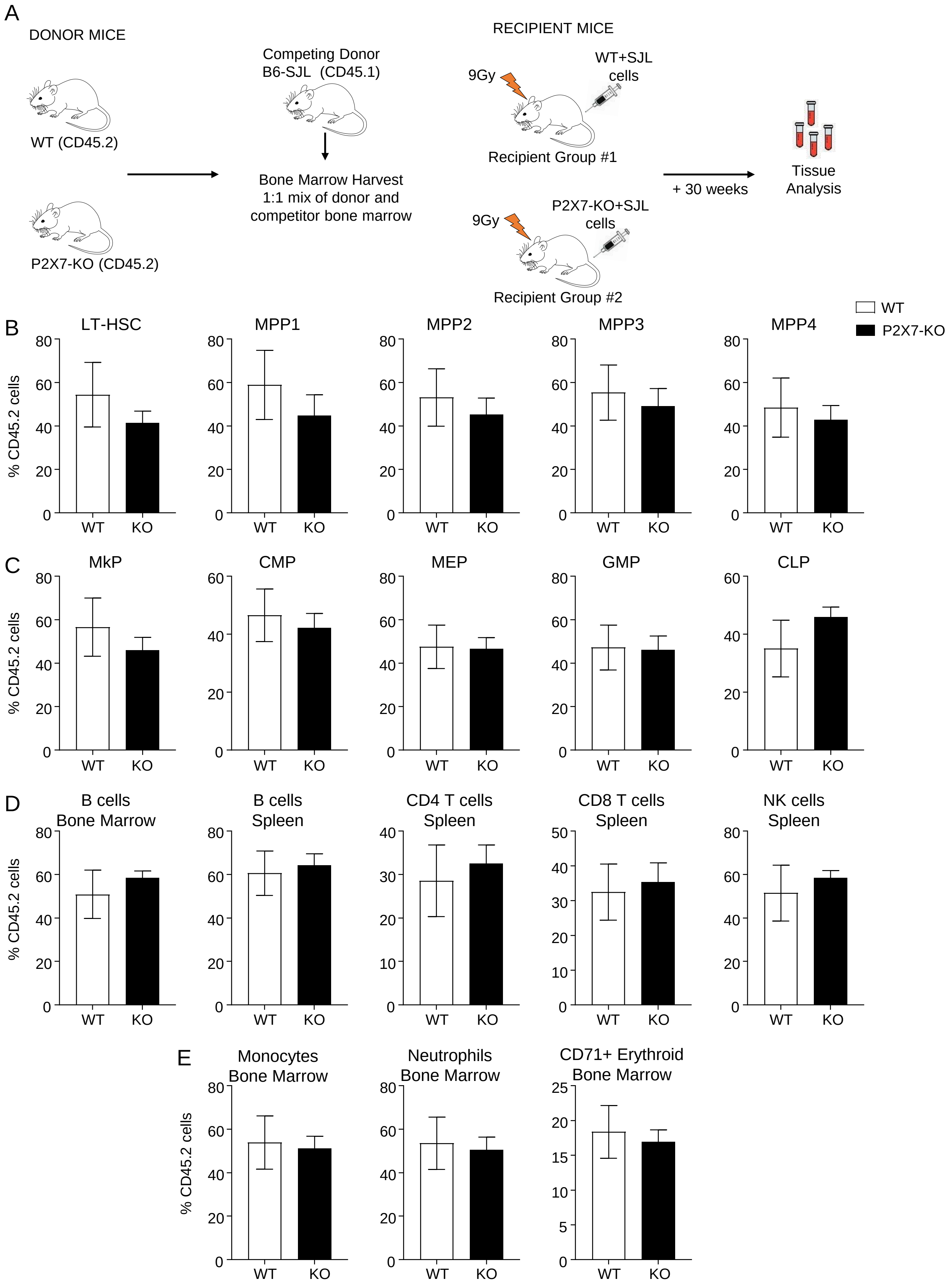
A
DONOR MICE
WT (CD45.2)
P2X7-KO (CD45.2)
Competing Donor
B6-SJL (CD45.1)
Bone Marrow Harvest
1:1 mix of donor and
competitor bone marrow
RECIPIENT MICE
9Gy
WT+SJL
cells
Recipient Group #1
9Gy
P2X7-KO+SJL
cells
Recipient Group #2
+ 30 weeks
Tissue
Analysis
WT
P2X7-KO
B
LT-HSC
MPP1
MPP2
MPP3
MPP4
% CD45.2 cells
C
MkP
CMP
MEP
GMP
CLP
D
B cells
Bone Marrow
B cells
Spleen
CD4 T cells
Spleen
CD8 T cells
Spleen
NK cells
Spleen
E
Monocytes
Bone Marrow
Neutrophils
Bone Marrow
CD71+ Erythroid
Bone Marrow
WT
KO

Figure S11. Assessing the role of P2X7 in the long-term HSC response and resistance to sub-lethal irradiation, using a competitive bone marrow transplantation model. (Related to Figure 7B).

Representative flow cytometry density plots showing the relative contribution of WT and P2X7-KO CD45.2^{+} cells, in direct competition with CD45.1^{+} B6-SJL cells, in the bone marrow transplantation model illustrated in Figure 7A. Briefly, bone marrow from P2X7-KO or WT mice was mixed with CD45.1-marked competitor bone marrow in a 1:1 ratio, and reconstituted into two independent groups of lethality irradiated recipients. After full reconstitution, the recipient mice were subjected to a sub-lethal irradiation at 3.5Gy, allowed to recover over a 30 week period, and subsequently analyzed by flow cytometry, measuring the frequency of CD45.2^{+} and CD45.1^{+} cells within each cell population, with 3-5 mice analyzed per group. The presented plots are for LT-HSCs, gated as Lin^{-}cKit^{+}Sca1^{+} followed by CD150^{+}CD48^{-}CD34^{-}Flt3^{-} cells. Bone marrow of control CD45.2^{+} B6 and CD45.1^{+} B6-SJL mice is analyzed in the top panel, and representative chimeric mice are analyzed at the bottom. The average cell frequency within each gate for all experimental mice in the group is presented as mean ± SD.

Figure S11

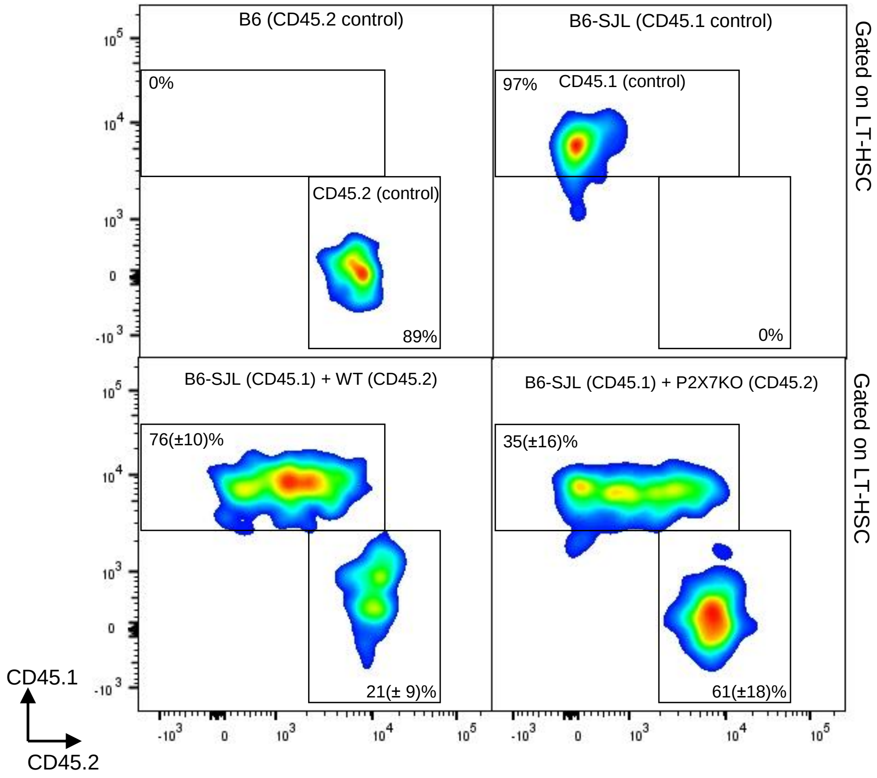
B6 (CD45.2 control)
0%
CD45.2 (control)
89%
B6-SJL (CD45.1 control)
97%
CD45.1 (control)
0%
Gated on LT-HSC
B6-SJL (CD45.1) + WT (CD45.2)
76(±10)%
21(± 9)%
B6-SJL (CD45.1) + P2X7KO (CD45.2)
35(±16)%
61(±18)%
Gated on LT-HSC
CD45.1
CD45.2

SUPPLEMENTAL DATA TABLES (LEGENDS)

Data tables S1-S3 are attached as separate files in Excel format.

Table S1. RNA-Seq transcriptional analyses of the response of wild type (WT) and p53-knockout (p53KO) murine HSPCs to whole body irradiation (IR). (Related to Figures 1, 3). **(A)** List of genes differentially expressed in WT-IR or p53KO-IR relative to control WT HSPCs, at fold change (FC) ≥2.0 and false discovery rate (FDR) ≤0.05; related to Figure 1C. Information provided for each gene includes: gene cluster and heatmap row number (Figure 1C), gene name, fold change, false discovery rate (FDR), and normalized counts per million. **(B)** Full list of genes expressed in HSPCs from WT and p53KO mice, with and without whole body irradiation; the same information as in (A) is provided for each gene. **(C)** Gene ontology enrichment analyses showing select enriched biological process (BP) terms for each cluster of differentially expressed genes from Figures 1C-D. **(D)** Normalized enrichment scores (NES) of 4,436 pre-established biological processes expression signatures used in the gene set enrichment analysis (GSEA) and depicted in Figure 1E and Figure 3. In each column, positive values indicate upregulation and negative values indicate downregulation in the comparisons.

Table S2. p53 ChIP-Seq from hematopoietic progenitor cells. (Related to Figures 2 and S1-2). **(A)** List of p53 DNA binding peaks identified in the hematopoietic progenitor cell line Ba/F3. Information provided for each p53 binding peak includes: row number on heatmap in Figure 2A, peak group in Figure 2A, peak genomic location, normalized read intensities ±100bp around the peak summit, distance to the nearest gene transcription start site (TSS), the identity of the nearest gene, whether the nearest gene is part of the GO pathways "regulation of myeloid cell differentiation", "IL-4 section" or "erythrocyte homeostasis", and whether this p53 binding peak is also identified in HPC7 cells or other published datasets from splenic B cells (7-Gy, 4hours, GSE71180) [29-30], bone marrow derived macrophages (BMDM, 6-Gy, GSE100963) [31], and mouse embryonic fibroblasts (MEFs, DOX treatment, GSE46240) [5]. **(B)** Spearman rank correlations between all pairwise comparisons for our p53 ChIP-Seq data with other published p53 or phospho-p53 ChIP-Seq datasets. Spearman correlations were calculated using the normalized tag densities (±100bp around peak summit) across the entire group of binding sites identified from all ChIP-Seq experiments. The p53 ChIP-Seq from Ba/F3 and HPC7 cells represent our datasets. The others are public datasets, downloaded and re-analyzed using our pipeline. These include: the p53 ChIP-Seq from splenic B and non-B cells (7-Gy, 4hours, GSE71180) [29-30], the phospho-p53 ChIP-Seq from bone marrow derived macrophages (BMDM, 6-Gy, GSE100963) [31], and the p53 ChIP-Seq from mouse embryonic fibroblasts (DOX-treatment, GSE46240) [5]; all datasets are from mouse. **(C)** Gene Ontology analysis of the genes nearest to each p53 DNA-binding peak, performed using the GREAT website (*http://great.stanford.edu/*) with basal plus extension option and searching for genes 2kb upstream, 2kb downstream, and 200kb in distal to each p53 peak. The -log10(binomial FDR) values are listed for each GO term; (related to Figure 2B). **(D)** Summary of RNA-Seq and

ChIP-Seq information for the genes encoding key regulators of HSC biology and hematopoiesis that have a p53 binding peak within 200kb to their gene TSS.

Table S3. Consolidation of the RNA-Seq and ChIP-Seq datasets characterizing the role of p53 in the transcriptional response of HSPCs to irradiation. (Related to Figures 2C-D).
(A) List of the putative p53-regulated genes, identified as RNA-Seq transcriptionally dysregulated genes within 10kb to a p53 binding peak. Information provided includes: gene name, information for the nearest p53 DNA binding peak from the ChIP-Seq data, gene dysregulation status from the RNA-Seq data, results of PubMed database search for publications that include the gene name together with the term "p53" in the title or in the abstract of the article, additional database searches for the characterization of the gene as a p53-target gene in KEGG pathway mmu04115 [38] and in 14 previous p53 ChIP/RNA-seq studies reviewed by Fischer M. et al. [4], and the detailed gene expression information and normalized CPMs for the genes. **(B)** List of 38 novel p53-target genes identified in our study, including the same information for each gene, as provided in (A). **(C)** Gene ontology enrichment analyses showing the enriched biological process (BP) terms for transcriptionally dysregulated genes that have p53 binding peaks within 10kb; related to Figure 2D. **(D)** Search result for gene-drug interactions using DGIdb (*https://www.dgidb.org/*) [39]. The gene names, drug names, and PubMed IDs of the reports are shown.

SUPPLEMENTAL METHODS TABLES S4-6

Table S4. ChIP-qPCR Primer Sequences.

Target Region	Forward Sequence	Reverse Sequence
Pomc<br>251bp upstream of TSS<br>(chr12:3,954,598-3,954,700)	aggcagatggacgcacataggtaa	tccacttagaactggacagaggct
P2rx7<br>621bp downstream of TSS<br>(chr5:123,094,541-123,094,626)	cccttgtgggctctgaatta	aggacctccaagatgctcta
Bbc3/PUMA<br>222bp upstream of TSS<br>(chr7:16,894,582-16,894,710)	tggtctgactttgtgtccct	gcttgacacactgacacact
Cdkn1a/p21<br>507bp downstream of short TSS<br>(chr17:29,231,224-29,231,320)	ccaaagcgtgagaatgaagctc	gctctgcgctaagctctagata

Table S5. Flow cytometry antibodies and other reagents used for immunophenotyping.
The following antibodies and reagents were used to acquire the data in Figures 5D-E and S3-S8.

Target	Fluorophore	Catalogue Number	Supplier
Apoptin	FITC	427401	BioLegend
B220	Biotinylated	103204	BioLegend
B220	APC-eFluor780	47-0452-82	eBioscience
BP1	FITC	11-5891-82	eBioscience
CD3	Biotinylated	100304	BioLegend
CD11b	Biotinylated	101204	BioLegend
CD16/32	Brilliant UV 737	612783	BD Biosciences
CD19	PE-Cy7	115520	BioLegend
CD24	eFluor450	48-0242-82	eBioscience
CD34	FITC	11-0341-85	eBioscience
CD41	Brilliant UV 395	564056	BD Biosciences
CD43	Brilliant UV 395	740224	BD Biosciences
CD48	PerCP-Cy5.5	103422	BioLegend
CD71	PE	113807	BioLegend
CD105	Pacific Blue	120411	BioLegend
CD150	PE-Cy7	115914	BioLegend
cKit	Brilliant Violet 650	135125	BioLegend
IgD	PerCP-Cy5.5	405710	BioLegend
IgM	PE	406508	BioLegend
P2X7	APC	148705	BioLegend
Sca1	APC-Cy7	108126	BioLegend
Streptavidin	Brilliant Violet 785	405249	BioLegend
TER119	Biotinylated	116204	BioLegend
TER119	PerCP-Cy5.5	116227	BioLegend
Viability Stain	v506	65-0866-18	eBioscience

Table S6. Flow cytometry antibodies and reagents used for bone marrow transplantation experiments.
The following antibodies and reagents were used to acquire the data in Figures 7 and S10-S11.

Target	Fluorophore	Catalogue Number	Supplier
B220	Biotinylated	103204	BioLegend
B220	Brilliant Violet 650	103241	BioLegend
BP1	FITC	11-5891-82	eBioscience
CD3	Biotinylated	100304	BioLegend
CD3	APC	100235	BioLegend
CD4	Brilliant Violet 785	100552	BioLegend
CD8	PerCP-Cy5.5	100734	BioLegend
CD11b	Biotinylated	101204	BioLegend
CD11b	eFluor450	48-0112-82	eBioscience
CD11c	Brilliant Violet 785	117336	BioLegend
CD19	PerCP-Cy5.5	115534	BioLegend
CD24	eFluor450	48-0242-82	eBioscience
CD25	PE	12-0251-81	eBioscience
CD34	Brilliant Violet 421	562608	BD Biosciences
CD34	FITC	11-0341-85	eBioscience
CD41	Brilliant UV 395	564056	BD Biosciences
CD43	Brilliant UV 395	740224	BD Biosciences
CD44	APC	17-0441-83	eBioscience
CD45.1	Brilliant UV 737	612811	BD Biosciences
CD45.2	PE-Cy7	109830	BioLegend
CD48	PerCP-Cy5.5	103422	BioLegend
CD69	FITC	11-0691-85	eBioscience
CD71	APC	113819	BioLegend
CD105	Pacific Blue	120411	BioLegend
CD150	PE	12-1502-82	eBioscience
CD150	Brilliant Violet 421	115925	BioLegend
CD16/CD32	FITC	11-0161-85	eBioscience
cKit	Brilliant Violet 650	135125	BioLegend
F4/80	FITC	35-4801-U100	Tonbo Biosciences
Flt3	PE	12-1351-83	eBioscience
IL7Ra	PE	12-1271-83	eBioscience
IgM	PE	406508	BioLegend
IgD	APC	405714	BioLegend
IgD	Pacific Blue	405712	BioLegend

Target	Fluorophore	Catalogue Number	Supplier
Ly6C	PE	128011	BioLegend
Ly6G	PerCP-Cy5.5	65-1276-U100	Tonbo Biosciences
MHCII	Brilliant Violet 650	107641	BioLegend
NK1.1	FITC	108706	BioLegend
Sca1	APC	17-5981-83	eBioscience
Streptavidin	PerCP-Cy5.5	405214	BioLegend
Streptavidin	Brilliant Violet 785	405249	BioLegend
TER119	Biotinylated	116204	BioLegend
Viability Stain	v506	65-0866-18	eBioscience
